# Communicable Diseases Prevalence among Refugees and Asylum Seekers: Systematic Review and Meta-Analysis

**DOI:** 10.3390/idr15020020

**Published:** 2023-03-31

**Authors:** Haitham Taha, Jo Durham, Simon Reid

**Affiliations:** 1 School of Public Health, The University of Queensland, Herston, QLD 4006, Australia; simon.reid@uq.edu.au; 2 School of Public Health and Social Work, The Queensland University of Technology, Kelvin Grove, QLD 4059, Australia; joanne.durham@qut.edu.au

**Keywords:** refugee, asylum seeker, communicable disease, tuberculosis, hepatitis, HIV, systematic review, meta-analysis

## Abstract

Background: Communicable diseases remain a significant global health issue. The increase in refugees and asylum seekers associated with conflicts may alter the burden of communicable diseases in host countries. We conducted a systematic review of the prevalence of TB, HBC, HCV, and HIV among refugees and asylum seekers by regions of asylum and origin. Methods: Four electronic databases were searched from initiation to the 25 December 2022. Prevalence estimates were pooled into a random-effect model and were stratified by the region of origin and asylum. Meta-analysis was conducted to explore the heterogeneity of the included studies. Results: The most-reported asylum region was The Americas, represented by the United States of America. Asia and the Eastern Mediterranean was the region of the most-reported origin. The highest reported prevalence of active TB and HIV was among African refugees and asylum seekers. The highest reported prevalence of latent TB, HBV and HCV was among Asian and Eastern Mediterranean refugees and asylum seekers. High heterogeneity was found regardless of the communicable disease type or stratification. Conclusion: This review provided insights about refugees’ and asylum seekers’ status around the world and attempted to connect refugees’ and asylum seekers’ distribution and the burden of communicable diseases.

## 1. Introduction

Currently, more than 65 million people have been forced to leave their home countries, and 23 million are refugees and asylum seekers [1]. Refugees and asylum seekers may hold a higher burden of communicable diseases such as tuberculosis (TB), hepatitis B virus (HBV), hepatitis C virus (HCV), and human immunodeficiency virus (HIV). For example, the prevalence of TB and measles among Syrian refugees and asylum seekers in host countries was found higher compared to the host population [2]. In Europe, many communicable diseases (CDs) were reported by refugees and asylum seekers [3,4,5,6]. It was estimated that 72% of new TB cases in England are related to refugees and asylum seekers [7]. Within refugee and asylum seeker settings, the CDs burden is mainly related to disease screening and control programs in the host and original countries. Additionally, route conditions reaching the asylum country aggravate the CDs burden [2]. Furthermore, Asylum seekers may spend protracted periods in transit with limited access to preventative health care and poor living conditions that may magnify CD transmission. For example, alerts were raised about the health status of Syrian asylum seekers who were isolated in deserted refugee camps because of poor access to health services and unfavorable health conditions [8]. Therefore, while the CDs burden is largely driven by refuge events and international crossing [3], CDs burden, surveillance and control will remain a source of concern, particularly in low-burden countries managing humanitarian crises [4,5,9,10].

We aim to estimate the prevalence of TB, HBV, HCV, and HIV in refugees and asylum seekers globally by region of origin and region of destinations (asylum).

## 2. Materials and Methods

The Preferred Reporting Items for Systematic Reviews and Meta-Analyses (PRISMA) guidelines were used (See Supplementary Materials S7).

We searched four bibliographic databases for indexing articles: PubMed, EMBASE, CINAHL, and Scopus. The searches commenced on 7 July 2018 and concluded on 20 November 2020 and were then updated on 25 December 2022. No limitations were set for time or country. In all four databases, we used a two-part keyword search strategy; in the first part, related to the population, we used ‘Refugee’ and another eight related terms to indicate the population of interest. In the second part, ‘Communicable disease’ and other related terms [9] were used. The preliminary search formula was Refugee* AND Communicable Disease (See Supplementary Materials S1–S4).

The keyword ‘Refugee’ was defined as any term used to indicate people fleeing to a safe place, especially those who flee to a foreign country to escape danger or persecution in their own country or habitual residence because of race, religion, or political belief [9]. ‘Communicable disease’ was defined as ‘an infectious disease transmissible (person to person) by direct or indirect contact (as by a vector)’ [9]. The definitions of TB (active or latent), hepatitis B, hepatitis C, and HIV diseases were drawn from each reference record included in the review as described by the original author of the study. If it was not clearly mentioned, the definition of the WHO was used to resolve any confusion and have a consistent description of the included diseases.

We used ‘MESH terms’ whenever suitable to capture all relevant studies. Specific terms related to our CDs of interest were hepatitis and HIV, and sexually transmitted diseases were included (See Supplementary Materials S1–S4). The clear status of each disease, whether it was the acute or past status of the disease, was not considered separately in this review; therefore, each included infection was analyzed based on the general status for the same disease.

Eligibility criteria (inclusion/exclusion)

Articles that fulfilled all the following characteristics were included in the systematic review.

1. Published, in English, specifically about ‘refugees’ and ‘asylum seekers’ of wars and conflicts.
2. Reported the prevalence of TB, HBV, HCV, and HIV by formal notification system diagnosis and as a systematic screening process.
3. Original research with a reportable prevalence denominator and numerator.

We excluded terms other than ‘Refugee’ or ‘asylum seeker’. For example, we excluded studies that referred to immigrants, migrants, or internally displaced people. Any non-empirical literature (letters to editors, commentaries, conference abstracts and transcripts, journalistic interviews, policy reports, and books) and any other studies with no full text available were also excluded. To minimize selection bias, we excluded studies conducted only for specific ages or genders such as children, pregnant women, pediatrics, or unaccompanied minors.

Study Selection (Screening Process)

The screening process conducted on all references is two-stepped:

1. Screening titles and abstracts (HT) and (SR).
2. Full texts screening and reading (HT) and (SR).

Title and abstract screening

We used Covidence [10], a web-based software platform, to manage title, abstract and full-text screening. All article titles and abstracts were screened according to the eligibility criteria by two reviewers (HT and SR). Judgment discrepancies in eligibility were resolved by team discussion (HT, SR, and JD) and consensus. If no clear exclusion criteria were decisive, we obtained the full text for references that were considered relevant by at least one reviewer.

Full-text screening and reading

After screening the title and abstract, we obtained full texts of included references. Independently, two reviewers (HT and SR) read the full text of the references and assessed their eligibility for inclusion. Conflicts regarding including references were resolved by discussions (HT, SR, and JD).

Data collection process (extraction)

We manually developed a data extraction sheet including the following items: study objectives (title) or research questions; years of publication; year of study; first author; population; country of origin; sampling method; sample coverage, instruments; study type; diseases under observation; sample size; prevalence; etc. (See Supplementary Materials S5). Wherever possible, data about specific population categories (i.e., Africa or Asia) were extracted and identified as part of our population of interest. We used the authors’ definitions of each disease status as stated in the studies.

Risk of bias in individual studies (critical appraisal)

We used Hoy et al.’s tool for quality assessment [11] to critically appraise the included studies (See Supplementary Materials S6). The risk of bias was assessed based on the questions included in the assessment tool and based on the following process: a score of 0 was given for questions answered as yes (low risk), and a score of 1 to those answered as no (high risk); then, the total number of scores was calculated. Consequently, the risk of bias was described according to the scale by calculating points out of 10: low risk scored 0–3; moderate risk scored 4–7, and high risk scored 8–10. Unanswerable questions were titled as not applicable (Table 1).

Prevalence records synthesis

We synthesized each prevalence record and noticed that a single study might include several prevalence records. Therefore, we extracted each prevalence record (comprised of denominator and numerator) in every included study, then assigned corresponding study information (author) for each extracted prevalence record and removed the duplicate records. We used extracted records to conduct the meta-analysis.

Meta-analysis

We used the Stata software package [12] to measure consistency (I^2^ test) and conduct a meta-analysis. We identified the numerator and denominator and conducted a random-effects meta-analysis to test the variability between included studies. We conducted pooled estimates under the supposition that each record of prevalence had dissimilar features and was measured differently. To investigate possible sources of heterogeneity, we undertook a subgroup analysis by stratifying the data by regions and CD type. We adopted the World Health Organization’s (WHO) definition of the regional grouping of countries to categorize studies by (See Supplementary Materials S8) [13,14,15].

Ethics review

Ethical clearance was not required for this systematic review.

## 3. Results

Study selection

**Figure 1:**
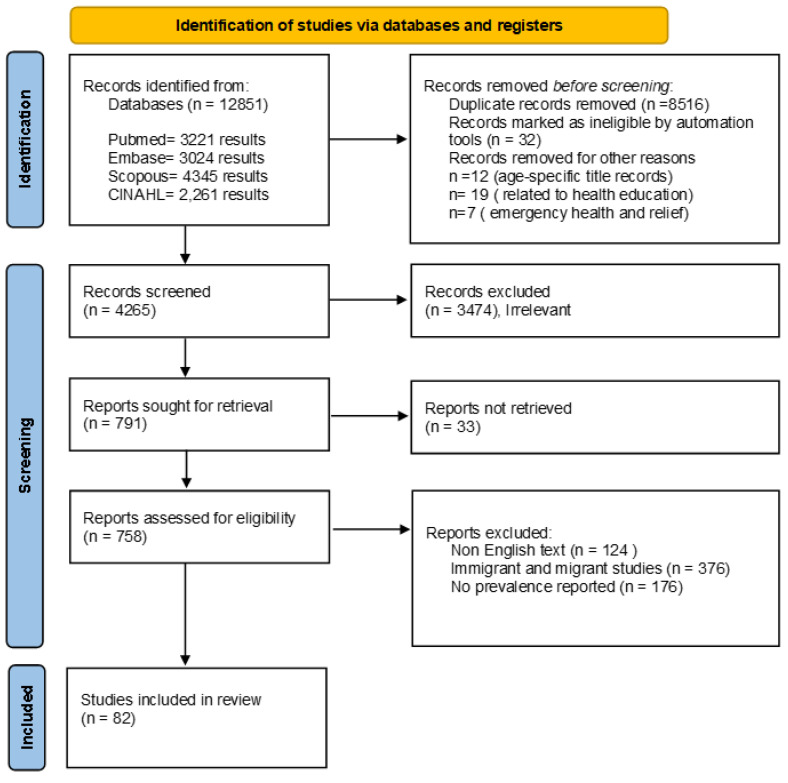
PRISMA flow chart study selection process.

We imported 12,851 references for screening. We screened 4265 unduplicated references, from which 791 were identified for full-text review and included 82 studies ([16,17,18,19,20,21,22,23,24,25,26,27,28,29,30,31,32,33,34,35,36,37,38,39,40,41,42,43,44,45,46,47,48,49,50,51,52,53,54,55,56,57,58,59,60,61,62,63,64,65,66,67,68,69,70,71,72,73,74,75,76,77,78,79,80,81,82,83,84,85,86,87,88,89,90,91,92,93,94,95,96]) (Figure 1).

Study characteristics

(A) Study topics and record synthesis

We included 82 studies representing 279 prevalence records. Studies conducted on TB, hepatitis (B and C) and HIV were as follows: 19 (24%), 20 (24%) and 2 (2.5%), respectively; the remaining studies (50%) conducted general health screening. We extracted 122 (44%), 99 (35%), 30 (11%) and 28 (10%) prevalence records of TB, HBV, HCV, and HIV, respectively. Most of the included studies were published by the United States of America (USA), 26 (32%); Italy, 11 (13%) and Australia, 7 (9%). We adopted the WHO’s definition of the regional grouping of countries to categories studies by regions; however, 26% of the records did not report country and were therefore put in a (Not specified) category. The most frequently reported regions of origin among the overall included records (n = 279) were as follows: Asia and the Eastern Mediterranean, 103 (37%), Africa, 63 (23%) and Europe, 34 (12%). For representation’s validity, results will be discussed as regions only (Table 2).

Meta-analysis and quality of included studies.

Generally, we found substantial variability among the included studies in terms of study objective, design, sample size, and Refugee and Asylum Seeker (RAS) regions of origin and periods (See Supplementary Materials S9). Consequently, it was difficult to apply the quality assessment protocol to included studies because we were unable to answer three questions (Not Applicable) of the tool for 96% of the included studies (78/82). However, most studies had a low to moderate risk of bias and internal validity had a low to moderate risk of bias, while external validity had a low risk of bias. Heterogeneity was significantly high in individual estimates among all included CD studies, even after stratification, and ranged between 85 and 99%. For further information regarding the meta-analysis, please refer to the Supplementary Material S9.

TB Prevalence

Most TB records (n = 122) were reported by the USA, 40 (33%), Australia and Germany, 14 (11%). We reported TB type as stated by the author of the included papers; however, if not specified, prevalence records were added to the ‘Unknown’ category (49%). A significant number of included papers (n = 52) did not report the used diagnostic tests to confirm TB diagnosis; however, they stated TB type. Therefore, we reported TB type as stated by the authors and did not report the diagnostic tests used because it was not comprehensively reported and was missing originally in a significant number of included studies.

Active TB

Active TB records (n = 29) were reported between the years 1981 and 2016, mostly by Germany and the United Kingdom (8/29) and in the years 2002 (9/29) and 2017 (8/29). However, the highest prevalence record of active TB (0.19) was in African RASs in Europe, and Belgium (19).

Latent TB

Latent TB records (n = 32) were reported between the years 1980 and 2018, mostly in the USA (15/32) and in the year 2009 (7/32). The highest prevalence of latent TB (0.70) was reported in Asian and Eastern Mediterranean RASs in the Western Pacific, Australia [61]. Among all statistically significant heterogeneous estimates, latent TB in RASs from Europe was insignificantly heterogeneous and had a prevalence estimate of <0.01 (95% CI: 0.00–0.01, I^2^ = 27%, *p*-value = 0.25). The following Table 3 presents the TB results.

HBV Prevalence

Included HBV records (n = 99) were reported between the years 1978 and 2018, mostly by the USA (39/99), Australia (13/99) and Italy (11/99) and in the years 2010 (15/99) and 2015 (11/99). The highest prevalence of HBV (0.48) was reported in Asian and Eastern Mediterranean RASs in the Western Pacific, Australia [18] (Table 4). A significant number of included papers (n = 41) did not report the used diagnostic tests to confirm HBV diagnosis. Therefore, we adopted the author’s definition of the HBV case and did not report the diagnostic tests used because it was not comprehensively reported and was missing originally in a significant number of included HBV studies.

HCV Prevalence

Included HCV records (n = 30) were reported between the years 1995 and 2018, mostly by Italy (6/30) and in the year 2015 (8/30). However, the highest prevalence record of HCV (0.31) was reported in Asian and Eastern Mediterranean RASs in Europe [75]. Among all statistically significant heterogeneous estimates, HCV heterogeneity was insignificant in RASs in Asia and the Eastern Mediterranean and Western Pacific, with prevalence estimates of 0.03 (95% CI: 0.01–0.05, I^2^ = 11%, *p*-value = 0.33) and 0.02 (95% CI: 0.01–0.02, I^2^ = 3%, *p*-value = 0.39), respectively (Table 5).

HIV Prevalence

Included HIV records (n = 28) were reported between the years 1995 and 2018, mostly in Italy and Germany (6/28) and in 2015 (6/28). The highest HIV prevalence (0.27) was reported in African RASs in Africa [38] (Table 6).

## 4. Discussion

This systematic review and meta-analysis include a significantly high coverage of studies (n = 82) compared to other reviews [97,98]. This is most likely explained due to the unrestricted search strategy conducted for this systematic review, to allow the capture of a wide range of studies from different origins and asylum countries. For example, other reviews [7,97] were conducted in specific settings only, such as country and time, and therefore included fewer studies. However, our review included a fewer number of studies compared to other conducted reviews that were restricted to time and location [86]. This could be explained by the discrepancy in eligibility criteria each review undertakes and the relevance/aims of each. Therefore, the aim of this review justifies the high yet reasonable number of included studies.

The overall heterogeneity in this review is high (I^2^ = 64–99) across all studies, even after stratification. This was evident in other reviews (24, 27, 28) conducted on a fewer number of studies [98] and/or restricted to a single country and time [97]. Heterogeneity is most likely explained as a real difference in CDs prevalence between RASs of different origin and asylum regions; as well, the epidemiologic status of CDs constantly changes over time within and between states, territories, and individuals, and like other diseases varies between regions and certain risk groups [99]. However, compared to other reviews that reported high heterogeneity levels (97–99%), our review reported less heterogeneity across included studies when stratified by region. The sub-analysis by regions generated low heterogeneous prevalence results in three regions, as follows: latent TB prevalence in RASs from Europe (<0.00, 95% CI: 0.00–0.01, I^2^ = 27.42, *p*-value = 0.25), HCV prevalence in RASs in Asia and the Eastern Mediterranean (0.03, 95% CI: 0.01–0.05, I^2^ = 11.70, *p*-value = 0.33) and the Western Pacific (0.02, 95% CI: 0.01–0.02, I^2^ = 3.75, *p*-value = 0.39). Therefore, considering I^2^ = 75% as a threshold for high heterogeneity, our meta-analysis showed relatively low levels of heterogeneity and might propose a promising method to direct and conduct future research in this field.

We found TB to be the most prevalent CD among RASs (19 studies and 122 records), particularly in Europe (50/122). This is consistent with other studies that reported RAS epidemiological data [100,101]. This might be linked to the past decade’s increase in the movement of RASs to European countries, which might be reflected in TB reporting. Since 2008, there has been a noticeable increase in the number of RASs hosted in Europe, which reached 5.2 million at the end of 2016 [102]. Therefore, TB prevalence among RASs may be explained by the continuous implementation of TB control programs such as screening at entry, by considering TB a major global health issue and a leading cause of mortality worldwide [97,103], particularly among RASs and international travelers from high-TB-burden countries. These results were similarly observed in a study conducted in Europe to evaluate migrants from the sub-Saharan countries [104]; the study found that almost 14% of the migrants had TB, which highlights again the importance of TB screening for early diagnosis and treatment in the country of asylum [104]. Furthermore, considering the health status of the migrants by screening at entry is very important for controlling the spread of TB among the local population, particularly as migrants are usually more exposed and more vulnerable than any other populations to diseases, whether chronic or infectious, despite healthy presentation at arrival [105].

We found differences in the prevalence ranges between active and latent TB. For example, we found a large disparity between the results of active TB between different regions, <0.01 and 0.6, which might indicate considerable variations between the included studies. However, we noticed that most of the reported prevalence is legitimately valid based on the methods used at that time. For example, the study that reported the highest active TB prevalence [39] used a TB notification system with a sample size (n = 8293) large enough to conclude the prevalence of active TB in RASs. Although this reported prevalence is inhomogeneous and considerably higher compared to other included records in our review [65], the results are reliable because there is a similarity in TB case notification rates between the reference country and our study in that same year. Yet, the study identifies 56 subjects lost to follow-up and assumes higher cases of active TB [39]. On the other hand, the reported maximum prevalence of latent TB between regions ranges between 0.50 and 0.70 is more consistent and considered higher than active TB, except for the Pacific (0.04) and The Americas (<0.01) (only one record for each). This may be explained by the diagnostic and reporting difficulties of active TB compared to latent TB. A patient’s medical history and a single test, a TB skin test, may be sufficient to diagnose and report latent TB. However, more complex procedures, such as chest radiographs and microbiology tests, are needed to diagnose active TB [106]. Therefore, most of the studies might have reported latent TB more consistently and frequently than active TB [101]. We found that RASs from the Asia and Eastern Mediterranean region had the highest HBV prevalence among RASs. These findings are consistent with other reviews [107,108] that associated RASs from East Asia with higher disease risks [107].

Based on regions of origin, HBV prevalence estimates ranged from <0.01 to 0.10. However, the highest reported HBV prevalence (0.48) was found in RASs from Asia and the Eastern Mediterranean who sought asylum in the Western Pacific, particularly Australia. These findings are most probably explained by the unprecedented refugee rates and the settling of RASs in low-HBV-burden countries such as Australia. This is further reflected in our findings in other regions such as Africa and Europe that have a comparably high prevalence: 0.37 and 0.27, respectively. Although estimated prevalence is not different among RASs by region of origin, it is subject to a high heterogeneity level (>94%) (See Table 4).

The finding of this review suggests that RASs from high or intermediate HCV prevalence regions present a high risk for HCV infection. We found that RASs from the Asia and Eastern Mediterranean region have the highest HCV prevalence (0.31). The HCV prevalence from our review is confined within acceptable prevalence ranges reported by other studies [98,108,109]. Globally, HCV prevalence is highest in Asia and the Eastern Mediterranean, Africa and Europe, ranging between 2% and 4%, but is less (<1.5%) in other regions such as Australia and The Americas. Although our findings were consistent with published regional findings, they are subject to the high heterogeneity of pooled estimates. Generally, the epidemiology of HCV is greatly variable within and between countries and regions because it is concentrated in certain risk groups that might alter over time. Therefore, the discrepancy might arise because of the wide range of HCV prevalence in individual countries in the same region [109,110]. In our study, the discrepancy in the reported and estimated prevalence may be due to the studies included in our review having a different risk profile of RASs, time, and characteristics of RASs.

We found that RASs from the African region reported the highest HIV prevalence (0.27). The findings are justified as most people living with HIV are in low- and middle-income countries, with an estimated 25 million living in Africa [111]. Our review suggests that RASs from high-/intermediate-HIV-prevalence regions, such as Africa, present a high risk for HCV infection. However, while previous reviews conducted on HIV in refugee settings were unable to establish sufficient evidence of HIV transmission [112], our results might be confounded only by the original global HIV distribution. This is further justified by the insufficient data at times of conflict and refugee crisis to conclude that RASs have a higher risk of HIV prevalence. However, our review provided HIV estimates consistent with other reviews conducted, particularly on RASs in African regions. For example, the prevalence of HIV infection ranged from 0.02 to 0.26 in RASs in African countries [112], matching with our results that range from 0.01 to 0.27, which therefore justifies our findings.

Although there is no consensus in the literature on how RASs could impact the epidemiology of local populations, some studies have discussed the range of risks that could emerge from hosting RASs from different geographical locations with certain infectious disease epidemiology. For example, while some RASs in Europe are found healthier than local populations, they are at higher risk for the main infectious diseases such as TB, HIV, and hepatitis [113]. This denotes that RASs could potentially be a source of infectious disease for local populations if there is direct or even indirect contact [114]. This highlights the need for the implementation of broad migrant screening campaigns to control infectious disease transmission from such populations [113]. On the other hand, it was found that RAS population migration has played a major role in disease transmission by starting outbreaks and increasing the prevalence and incidence of infectious diseases in the country of asylum. Moreover, the RAS population is considered a main factor in introducing infectious diseases and increasing transmission potential with local populations [114]. For example, in North America, the role of the migrant population, such as refugees, in disease transmission ranged from incubating infections such as hepatitis A, reactivating latent diseases such as TB, or bearing an increased burden of the diseases [114]. This highlights the challenges that might be faced by local health authorities and the need to manage illnesses and diseases attributed to global factors that are beyond the national disease control strategies and management plans. The type of risk that emerges from the RAS populations will have effects that grow in clinical, control and management nature, which will identify RASs as an important risk group that continuously poses new challenges as more RASs arrive from new geographical regions with variable infectious disease epidemiology and demography [115].

### Limitations

Several limitations were observed in our review. Adjusting for RASs’ characteristics was practically challenging and was therefore reflected in homogeneity levels and findings [96,98,106]. For example, we attempted to report countries of origin/asylum; however, we noticed discrepancies across the literature regarding countries/regions reported without standard geographical classification. Therefore, we chose to report our results according to the WHO’s geo-regional definitions. Overall, 25% did not report geographical origin and were therefore grouped as ‘Not specified’. Failure to report geographical regions affected the validity of the findings and the homogeneity. Additionally, prevalence records were not always reported clearly by the authors and needed manual calculation. RAS definitions were not clear and were indistinguishable from similar populations such as migrants [7]. This led to the exclusion of many studies that did not clearly define RASs, and therefore a loss of data [7]. Additionally, studies were not always disease-specific but presented a general description of CDs (n = 40/82). For example, 62/122 (51%) of TB records failed to specify the TB type (active or latent). The overall quality of the risk of bias assessment is affected by including a wide range of studies and study designs [89,96].

This review did not provide a separate analysis of each disease status, i.e., whether acute or past, but each included infection was analyzed based on the general status for the same disease. This could have created minor discrepancy in the results compared to other studies that considered the disease status as active vs. latent, for example [116]. Consequently, the results of this review might be subject to a bias and inaccuracies in the pooled prevalence [117]. However, considering the aim of this review, this expected bias might have little effect on looking at the overall estimates of the diseases in refugee populations at the countries of asylum, although estimating the status of each disease precisely would provide more confidence and rigor to the review.

## 5. Conclusions

This review provides insights into RAS status around the world and the associated disease burden across regions with RAS distribution. We identified limited evidence on TB, HBV, HCV, and HIV prevalence among RASs and reported CD prevalence based on regions of origin and asylum, which could necessitate future research. Tracking CD prevalence among RASs is an important element of any disease control strategy in hosting countries, particularly countries managing humanitarian crises. Therefore, these results may provide useful data to inform the policy development for CD screening, detection, and control in RAS settings.

We recommend further research on the national and international levels. Valid data are needed, particularly regarding RASs, along with reliable study designs. The definition of RASs should be clear and consistent across all conducted studies, to delineate between different groups and aid in better research.

## Figures and Tables

**Table 1:** Risk of bias scale of included studies.

Calculated Risk Score	Correspondent Risk
0–3	Low risk
4–7	Moderate risk
7–10	High Risk

**Table 2:** Regions of origins of refugees and asylum seekers, reported in records (n = 279).

Regions of Origin	Number (%)
Asia and Eastern Mediterranean	103 (37)
Africa	63 (23)
Europe	33 (12)
The Americas	5 (2)
Western Pacific	3 (1)
Not Specified	72 (26)
Total	279 (100)

**Table 3:** Tuberculosis prevalence estimates, 95% confidence interval, heterogeneity level (I^2^), *p*-value, records, median, minimum, and maximum reported prevalence among refugees and asylum seekers by type, regions of origins and asylum.

Tuberculosis	Estimated Prevalence				Records	Reported Prevalence		
	Prevalence	95% CI	I^2^	*p*-Value	(n = 122)	Median	Min	Max
Active TB								
Regions of Origin								
Western Pacific	0.02	0.01–0.09	N/A *	N/A	1	0.02	0.02	0.02
Not specified	0.00	0.00–0.00	91	0.00	6	0.02	0.00	0.05
Asia and Eastern Mediterranean	0.03	0.02–0.05	98	0.00	10	0.01	0.00	0.17
Europe	0.00	0.00–0.01	81	0.00	5	0.01	0.00	0.05
Africa	0.01	0.01–0.02	94	0.00	6	0.01	0.03	0.19
The Americas	0.00	0.00	N/A	N/A	1	<0.00	<0.00	<0.00
Regions of Asylum								
Europe	0.00	0.00–0.00	95	0.00	24	0.01	0.00	0.19
The Americas	0.19	0.04–0.42	98	0.00	3	0.08	0.04	0.17
Western Pacific	0.02	0.01–0.06	N/A	N/A	1	0.02	0.02	0.02
Asia and Eastern Mediterranean	0.01	0.01	N/A	N/A	1	0.01	0.01	0.01
Total Active					29			
Latent TB								
Regions of Origin								
Western Pacific	0.04	0.04–0.05	N/A	N/A	1	0.04	0.04	0.04
Not specified	0.40	0.27–0.53	98	0.00	8	0.44	0.14	0.55
Asia and Eastern Mediterranean	0.14	0.09–0.21	99	0.00	11	0.11	0.01	0.70
Europe	0.00	0.00–0.01	27	0.25	4	0.04	0.02	0.50
Africa	0.29	0.07–0.58	99	0.00	7	0.43	0.01	0.64
The Americas	0.00	0.00	N/A	N/A	1	<0.00	<0.00	<0.00
Regions of Asylum								
Europe	0.15	0.03–0.32	98	0.00	8	0.07	0.02	0.48
The Americas	0.16	0.13–0.19	99	0.00	22	0.20	0.00	0.64
Western Pacific	0.60	0.56–0.65	N/A	N/A	2	0.63	0.55	0.70
Total Latent					32			
Unknown TB type								
Regions of Origin								
Africa	0.21	0.14–0.29	99	0.00	12	0.25	0.00	0.72
The Americas	0.00	0.00	N/A	N/A	2	0.00	0.00	0.00
Asia and Eastern Mediterranean	0.23	0.17–0.29	99	0.00	29	0.29	0.00	0.69
Europe	0.12	0.00–0.34	99	0.00	5	0.04	0.01	0.49
Not specified	0.19	0.10–0.30	99	0.00	12	0.22	0.00	0.65
Western Pacific	0.06	0.05–0.06	N/A	N/A	1	0.06	0.06	0.06
Regions of Asylum								
Europe	0.08	0.05–0.11	99	0.00	18	0.03	0.00	0.65
Western Pacific	0.21	0.11–0.34	99	0.00	13	0.25	0.02	0.42
The Americas	0.26	0.022–0.30	99	0.00	23	0.40	0.00	0.72
Africa	0.00	0.00	N/A	N/A	1	0.00	0.00	0.00
Asia and Eastern Mediterranean	0.31	0.02–0.73	99	0.00	5	0.35	0.00	0.69
Total Unknown					61			

* Not available due to low number of records.

**Table 4:** Hepatitis B virus prevalence estimates, 95% confidence interval, heterogeneity level (I^2^), *p*-value, records, median, minimum, and maximum reported prevalence among refugees and asylum seekers.

Hepatitis B Virus	Estimated Prevalence (Meta-Analysis)				Records	Reported Prevalence		
	Prevalence	95% CI	I^2^	*p*-Value	(n = 99)	Median	Min	Max
Regions of Origin								
Africa	0.08	0.07–0.10	94	0.00	23	0.08	0.01	0.37
Not Specified	0.08	0.06–0.11	97	0.00	22	0.06	0.00	0.45
Asia and Eastern Mediterranean	0.08	0.06–0.10	98	0.00	40	0.06	0.00	0.48
Europe	0.07	0.04–0.11	98	0.00	13	0.03	0.00	0.27
The Americas	0.01	0.00–0.02	N/A	N/A	1	0.00	0.00	0.00
Regions of Asylum								
Africa	0.14	0.12–0.17	N/A *	N/A	2	0.14	0.13	0.14
Asia and Eastern Mediterranean	0.06	0.03–0.05	93	0.00	10	0.05	0.00	0.21
The Americas	0.06	0.05–0.08	98	0.00	44	0.65	0.00	0.38
Western Pacific	0.11	0.08–0.14	92	0.00	15	0.09	0.02	0.48

* Not available due to low number of records.

**Table 5:** Hepatitis C virus prevalence estimates, 95% confidence interval, heterogeneity level (I^2^), *p*-value, records, median, minimum, and maximum reported prevalence among refugees and asylum seekers.

Hepatitis C Virus	Estimated Prevalence (Meta-Analysis)				Records	Reported Prevalence		
	Prevalence	95% CI	I^2^	*p*-Value	(n = 30)	Median	Min	Max
Regions of Origin								
Africa	0.03	0.01–0.07	91	0.00	5	0.03	0.00	0.10
Asia and Eastern Mediterranean	0.03	0.01–0.06	79	0.00	7	0.02	0.01	0.31
Europe	0.04	0.00–0.09	93	0.00	4	0.01	0.00	0.27
Not Specified	0.01	0.01–0.02	70	0.00	14	0.01	0.00	0.12
Regions of Asylum								
Africa	0.01	0.01–0.03	N/A *	N/A	2	0.01	0.00	0.03
Asia and Eastern Mediterranean	0.03	0.01–0.05	11	0.33	4	0.05	0.02	0.05
Europe	0.03	0.02–0.06	90	0.00	14	0.02	0.00	0.31
The Americas	0.01	0.00–0.02	64	0.04	4	0.01	0.00	0.01
Western Pacific	0.02	0.01–0.02	3	0.39	6	0.02	0.01	0.03

* Not available due to low number of records.

**Table 6:** Human immunodeficiency virus prevalence estimates, 95% confidence interval, heterogeneity level (I^2^), *p*-value, records, median, minimum, and maximum reported prevalence among refugees and asylum seekers.

HIV	Estimated Prevalence (Meta-Analysis)				Records	Reported Prevalence		
	Prevalence	95% CI	I^2^	*p*-Value	(n = 28)	Median	Min	Max
Regions of Origin								
Africa	0.04	0.03–0.06	94	0.00	10	0.03	0.01	0.27
Not Specified	0.02	0.01–0.04	96	0.00	10	0.01	0.00	0.11
Asia and Eastern Mediterranean	0.00	0.00–0.01	80	0.00	6	0.00	0.00	0.03
Europe	0.00	0.00–0.00	N/A *	N/A	2	0.05	0.00	0.11
Regions of Asylum								
Africa	0.07	0.05–0.09	N/A	N/A	2	0.15	0.03	0.27
Europe	0.02	0.01–0.03	98	0.00	13	0.01	0.00	0.11
The Americas	0.04	0.02–0.07	83	0.00	7	0.05	0.00	0.11
Western Pacific	0.01	0.00–0.03	97	0.00	6	0.01	0.00	0.03

* Not available due to low number of records.

## Data Availability

Not applicable.

## Supplementary Materials

The following supporting information can be downloaded at: https://www.mdpi.com/article/10.3390/idr15020020/s1. S1: PubMed search strategy history, S2: Scopus search strategy history, S3: Embase search strategy history, S4: Cinahl search strategy history, S5: Systematic review data extraction sheet, S6: Risk of Bias Tool, Hoy et al., 2012, S7: PRISMA 2009 Checklist, S8: WHO Estimates of TB incidence, HBV and HCV prevalence, and HIV-people living, based, 2022, S9: Forest plots of the meta-analysis by the disease.

## References

[1] UNHCR Figures at a Glance. https://www.unhcr.org/figures-at-a-glance.html.

[2] United Nations Development Programme (2023). Needs Assessment Review of the Impact of the Syrian Crisis on Jordan.

[3] Nyiri P., Eling J. (2012). A specialist clinic for destitute asylum seekers and refugees in London. Br. J. Gen. Pract..

[4] van Berlaer G., Bohle Carbonell F., Manantsoa S., de Béthune X., Buyl R., Debacker M., Hubloue I. (2016). A refugee camp in the centre of Europe: Clinical characteristics of asylum seekers arriving in Brussels. BMJ Open.

[5] Hertting O., Luthander J., Giske C.G., Bennet R., Eriksson M. (2021). Acute infection as cause of hospitalization of asylum-seeking children and adolescents in Stockholm, Sweden 2015–2016. Eur. J. Pediatr..

[6] Kühne A., Gilsdorf A. (2016). Infectious disease outbreaks in centralized homes for asylum seekers in Germany from 2004–2014. Bundesgesundheitsblatt Gesundh. Gesundh..

[7] Clark R.C., Mytton J. (2007). Estimating infectious disease in UK asylum seekers and refugees: A systematic review of prevalence studies. J. Public Health.

[8] Amnesty International (2016). Syria-Jordan Border: 75,000 Refugees Trapped in Desert No Man’s Land in Dire Conditions. https://www.amnesty.org/en/latest/news/2016/09/syria-jordan-border-75000-refugees-trapped-in-desert-no-mans-land-in-dire-conditions/.

[9] PubMed. https://www.ncbi.nlm.nih.gov/pubmed/.

[10] Covidence-Better Systematic Review Management. https://www.covidence.org/.

[11] Hoy D., Brooks P., Woolf A., Blyth F., March L., Bain C., Baker P., Smith E., Buchbinder R. (2012). Assessing risk of bias in prevalence studies: Modification of an existing tool and evidence of interrater agreement. J. Clin. Epidemiol..

[12] Stata (2023). Statistical Software for Data Science|Stata. https://www.stata.com/.

[13] WHO (2017). Global Hepatitis Report. https://www.who.int/publications/i/item/9789241565455.

[14] WHO (2022). Global Tuberculosis Programme. Global Tuberculosis Reports. https://www.who.int/teams/global-tuberculosis-programme/tb-reports.

[15] WHO (2023). Global HIV Programme. HIV Data and Statistics. https://www.who.int/teams/global-hiv-hepatitis-and-stis-programmes/hiv/strategic-information/hiv-data-and-statistics.

[16] Martin J.A., Mak D.B. (2006). Changing faces: A review of infectious disease screening of refugees by the Migrant Health Unit, Western Australia in 2003 and 2004. Med. J. Aust..

[17] Tengve B. (1968). A tuberculosis survey in Mwesi Highland Settlement for refugees in Tanzania. E. Afr. Med. J..

[18] Christopher P.J., Millsom R.H., Bailey K.F. (1978). Indo-Chinese Refugees. Med. J. Aust..

[19] Jones M.J., Thompson J.H., Brewer N.S. (1980). Infectious diseases of Indochinese refugees. Mayo. Clin. Proc..

[20] (1981). Michigan department of social services ranks leading diagnoses among refugees. Mich. Med..

[21] Catanzaro A., Moser R.J. (1982). Health status of refugees from Vietnam, Laos, and Cambodia. Jama.

[22] Chadwick R.G., Hall A.J., Davidson I., Bull F.G., Wright R. (1982). Hepatitis B among Indochinese refugees in Great Britain. Postgrad. Med. J..

[23] Barry M., Craft J., Coleman D., Coulter H.O., Horwitz R. (1983). Clinical findings in Southeast Asian refugees. Jama.

[24] Engebretsen B., Knight A., Shah R. (1984). Hepatitis B in Southeast Asian refugees in Iowa. Iowa Med..

[25] Judson F.N., Lince D.M., Anders B.J., Tapy J.M., Le Van D., Cohn D.L., Kicera T.J. (1984). Health status of Southeast Asian refugees. West. J. Med..

[26] McCaw B.R., DeLay P. (1985). Demographics and disease prevalence of two new refugee groups in San Francisco. The Ethiopian and Afghan refugees. West. J. Med..

[27] Parenti D.M., Lucas D., Lee A., Hollenkamp R.H. (1987). Health status of Ethiopian refugees in the United States. Am. J. Public Health.

[28] Zellweger J.P., Vejdovsky R. (1988). Tuberculosis among refugees: Study of a population screening at the Tuberculosis Clinic in Lausanne (Switzerland) between 1983 and 1988. Bull. Int. Union Against Tuberc. Lung Dis..

[29] Chow R.T., Krumholtz S. (1989). Health screening of a RI Cambodian refugee population. Rhode Isl. Med. J..

[30] Sutter R.W., Haefliger E. (1990). Tuberculosis morbidity and infection in Vietnamese in Southeast Asian refugee camps. Am. Rev. Respir. Dis..

[31] Santantonio T., Lo Caputo S., Germinario C., Squarcione S., Greco D., Laddago V., Pastore G. (1993). Prevalence of hepatitis virus infections in Albanian refugees. Eur. J. Epidemiol..

[32] Bisrat F., Berhane Y., Mamo A., Asefa E. (1995). Morbidity pattern among refugees in Eastern Ethiopia. E. Afr. Med. J..

[33] Bos P., Steele A.D., Peenze I., Aspinall S. (1995). Sero-prevalence to hepatitis B and C virus infection in refugees from Mozambique in southern Africa. E. Afr. Med. J..

[34] Dalekos G.N., Zervou E., Karabini F., Tsianos E.V. (1995). Prevalence of viral markers among refugees from southern Albania: Increased incidence of infection with hepatitis A, B and D viruses. Eur. J. Gastroenterol. Hepatol..

[35] Hurie M.B., Gennis M.A., Hernandez L.V., Dindzans V.J., Davis J.P. (1995). Prevalence of hepatitis B markers and measles, mumps, and rubella antibodies among Jewish refugees from the former Soviet Union. JAMA.

[36] Van Rensburg E.J., Lemmer H.R., Joubert J.J. (1995). Prevalence of viral infections in Mozambican refugees in Swaziland. E. Afr. Med. J..

[37] Gjerdingen D.K., Lor V. (1997). Hepatitis B status of Hmong patients. J. Am. Board. Fam. Pract..

[38] Maayan S., Marks N., Viterbro A., Zeide Y., Morag A., Neil L., Strauss N., Shapiro M. (1997). HIV infection and susceptibility to epidemic bacterial infections among Rwandan refugees. Int. J. Infect. Dis..

[39] Van den Brande P., Uydebrouck M., Vermeire P., Demedts M. (1997). Tuberculosis in asylum seekers in Belgium. VRGT (Flemish Lung and Tuberculosis Association). Eur. Respir. J..

[40] Litch J.A., Shackleton J.R., Bishop R.A. (1998). Prevalence of hepatitis B infection among Tibetan refugees in northern India. Trop. Doct..

[41] Chironna M., Germinario C., Lopalco P.L., Carrozzini F., Quarto M. (2001). Prevalence of hepatitis virus infections in Kosovar refugees. Int. J. Infect. Dis..

[42] Chironna M., Germinario C., Lopalco P.L., Quarto M., Barbuti S. (2000). HBV, HCV and HDV infections in Albanian refugees in Southern Italy (Apulia region). Epidemiol. Infect..

[43] Callister M.E., Barringer J., Thanabalasingam S.T., Gair R., Davidson R.N. (2002). Pulmonary tuberculosis among political asylum seekers screened at Heathrow Airport, London, 1995–1999. Thorax.

[44] Hobbs M., Moor C., Wansbrough T., Calder L. (2002). The health status of asylum seekers screened by Auckland Public Health in 1999 and 2000. N. Z. Med. J..

[45] Kelly P.M., Scott L., Krause V.L. (2002). Tuberculosis in east timorese refugees: Implications for health care needs in East Timor. Int. J. Tuberc. Lung Dis..

[46] Lifson A.R., Thai D., O’Fallon A., Mills W.A., Hang K. (2002). Prevalence of tuberculosis, hepatitis B virus, and intestinal parasitic infections among refugees to Minnesota. Public Health Rep..

[47] Ullah S., Shah S.H., Rehman A.U., Kamal A., Begum N. (2002). Tuberculous lymphadenitis in Afghan refugees. J. Ayub Med. Coll. Abbottabad.

[48] Chironna M., Germinario C., Lopalco P.L., Carrozzini F., Barbuti S., Quarto M. (2003). Prevalence rates of viral hepatitis infections in refugee Kurds from Iraq and Turkey. Infection.

[49] Marras T.K., Wilson J., Wang E.E., Avendano M., Yang J.W. (2003). Tuberculosis among Tibetan refugee claimants in Toronto: 1998 to 2000. Chest.

[50] Roussos A., Goritsas C., Pappas T., Spanaki M., Papadaki P., Ferti A. (2003). Prevalence of hepatitis B and C markers among refugees in Athens. World J. Gastroenterol..

[51] Rysstad O.G., Gallefoss F. (2003). TB status among Kosovar refugees. Int. J. Tuberc. Lung Dis..

[52] van Burg J.L., Verver S., Borgdorff M.W. (2003). The epidemiology of tuberculosis among asylum seekers in The Netherlands: Implications for screening. Int. J. Tuberc. Lung Dis..

[53] LoBue P.A., Moser K.S. (2004). Screening of immigrants and refugees for pulmonary tuberculosis in San Diego County, California. Chest.

[54] McLeod A., Reeve M. (2005). The health status of quota refugees screened by New Zealand’s Auckland Public Health Service between 1995 and 2000. N. Z. Med. J..

[55] Shah B.K., Bhattacharya S., Parija S.C. (2005). Seroprevalence of hepatitis B virus among Bhutanese refugees residing in Nepal. Kathmandu Univ. Med. J..

[56] Quddus A., Luby S.P., Jamal Z., Jafar T. (2006). Prevalence of hepatitis B among Afghan refugees living in Balochistan, Pakistan. Int. J. Infect. Dis..

[57] Tiong A.C., Patel M.S., Gardiner J., Ryan R., Linton K.S., Walker K.A., Scopel J., Biggs B.A. (2006). Health issues in newly arrived African refugees attending general practice clinics in Melbourne. Med. J. Aust..

[58] Choi C.M., June J.H., Kang C.I., Park J.T., Oh S.Y., Lee J.B., Lee C.H., Yim J.J., Kim H.J. (2007). Tuberculosis among dislocated North Koreans entering Republic of Korea since 1999. J. Korean Med. Sci..

[59] Pottie K., Janakiram P., Topp P., McCarthy A. (2007). Prevalence of selected preventable and treatable diseases among government-assisted refugees: Implications for primary care providers. Can. Fam. Physician.

[60] Ugwu C., Varkey P., Bagniewski S., Lesnick T. (2008). Sero-epidemiology of hepatitis B among new refugees to Minnesota. J. Immigr. Minor. Health.

[61] Chaves N.J., Gibney K.B., Leder K., O’Brien D.P., Marshall C., Biggs B.A. (2009). Screening practices for infectious diseases among Burmese refugees in Australia. Emerg. Infect. Dis..

[62] Liu Y., Weinberg M.S., Ortega L.S., Painter J.A., Maloney S.A. (2009). Overseas screening for tuberculosis in U.S.-bound immigrants and refugees. N. Engl. J. Med..

[63] Museru O., Franco-Paredes C. (2009). Epidemiology and clinical outcomes of hepatitis B virus infection among refugees seen at a U.S. travel medicine clinic: 2005–2008. Travel. Med. Infect. Dis..

[64] Tafuri S., Prato R., Martinelli D., Melpignano L., De Palma M., Quarto M., Germinario C. (2010). Prevalence of Hepatitis B, C, HIV and syphilis markers among refugees in Bari, Italy. BMC Infect. Dis..

[65] Pacifici L.E., Riccardo F., Russo G., Miccoli G.A., Vullo V. (2010). Screening for tuberculosis among asylum seekers: Experience from an immigration centre in Central Italy and literature review. G. Ital. Di Mal. Trop..

[66] Rein D.B., Lesesne S.B., O’Fallon A., Weinbaum C.M. (2010). Prevalence of hepatitis B surface antigen among refugees entering the United States between 2006 and 2008. Hepatology.

[67] Johnston V., Smith L., Roydhouse H. (2012). The health of newly arrived refugees to the Top End of Australia: Results of a clinical audit at the Darwin Refugee Health Service. Aust. J. Prim. Health.

[68] Paxton G.A., Sangster K.J., Maxwell E.L., McBride C.R., Drewe R.H. (2012). Post-arrival health screening in Karen refugees in Australia. PLoS ONE.

[69] Stauffer W.M., Painter J., Mamo B., Kaiser R., Weinberg M., Berman S. (2012). Sexually transmitted infections in newly arrived refugees: Is routine screening for Neisseria gonorrheae and Chlamydia trachomatis infection indicated?. Am. J. Trop. Med. Hyg..

[70] Chai S.J., Davies-Cole J., Cookson S.T. (2013). Infectious disease burden and vaccination needs among asylees versus refugees, district of columbia. Clin. Infect. Dis..

[71] Köse Ş., Kuzucu L., Gözaydın A., Yılmazer T. (2015). Prevalence of hepatitis B and C viruses among asylum seekers in Izmir. J. Immigr. Minor. Health.

[72] Lacourse S., Rybak N., Lewis C., Gartman J., Larkin J., McLaughlin S., Toll E.T. (2013). Health screening of newly resettled refugees in a primary care setting. R. I. Med. J..

[73] Bennett R.J., Brodine S., Waalen J., Moser K., Rodwell T.C. (2014). Prevalence and treatment of latent tuberculosis infection among newly arrived refugees in San Diego County, January 2010–October 2012. Am. J. Public Health.

[74] Bertelsen N.S., Selden E., Krass P., Keatley E.S., Keller A. (2018). Primary Care Screening Methods and Outcomes for Asylum Seekers in New York City. J. Immigr. Minor. Health.

[75] Coppola N., Alessio L., Gualdieri L., Pisaturo M., Sagnelli C., Caprio N., Maffei R., Starace M., Angelillo I.F., Pasquale G. (2015). Hepatitis B virus, hepatitis C virus and human immunodeficiency virus infection in undocumented migrants and refugees in southern Italy, January 2012 to June 2013. Eurosurveillance.

[76] Meier V., Artelt T., Cierpiol S., Gossner J., Scheithauer S. (2016). Tuberculosis in newly arrived asylum seekers: A prospective 12 month surveillance study at Friedland, Germany. Int. J. Hyg. Environ. Health.

[77] Mixson-Hayden T., Lee D., Ganova-Raeva L., Drobeniuc J., Stauffer W.M., Teshale E., Kamili S. (2014). Hepatitis B virus and hepatitis C virus infections in United States-bound refugees from Asia and Africa. Am. J. Trop. Med. Hyg..

[78] Padovese V., Egidi A.M., Melillo T.F., Farrugia B., Carabot P., Didero D., Costanzo G., Mirisola C. (2014). Prevalence of latent tuberculosis, syphilis, hepatitis B and C among asylum seekers in Malta. J. Public. Health.

[79] Ravensbergen S.J., Lokate M., Cornish D., Kloeze E., Ott A., Friedrich A.W., van Hest R., Akkerman O.W., de Lange W.C., van der Werf T.S. (2016). High Prevalence of Infectious Diseases and Drug-Resistant Microorganisms in Asylum Seekers Admitted to Hospital; No Carbapenemase Producing Enterobacteriaceae until September 2015. PLoS ONE.

[80] Redditt V.J., Janakiram P., Graziano D., Rashid M. (2015). Health status of newly arrived refugees in Toronto, Ont: Part 1: Infectious diseases. Can. Fam. Physician.

[81] Russo G., Vita S., Miglietta A., Terrazzini N., Sannella A., Vullo V. (2016). Health profile and disease determinants among asylum seekers: A cross-sectional retrospective study from an Italian reception centre. J. Public Health.

[82] Ackermann N., Marosevic D., Hörmansdorfer S., Eberle U., Rieder G., Treis B., Berger A., Bischoff H., Bengs K., Konrad R. (2018). Screening for infectious diseases among newly arrived asylum seekers, Bavaria, Germany, 2015. Eurosurveillance.

[83] Alberer M., Malinowski S., Sanftenberg L., Schelling J. (2018). Notifiable infectious diseases in refugees and asylum seekers: Experience from a major reception center in Munich, Germany. Infection.

[84] Bloch-Infanger C., Bättig V., Kremo J., Widmer A.F., Egli A., Bingisser R., Battegay M., Erb S. (2017). Increasing prevalence of infectious diseases in asylum seekers at a tertiary care hospital in Switzerland. PLoS ONE.

[85] Buonfrate D., Gobbi F., Marchese V., Postiglione C., Badona Monteiro G., Giorli G., Napoletano G., Bisoffi Z. (2018). Extended screening for infectious diseases among newly-arrived asylum seekers from Africa and Asia, Verona province, Italy, April 2014 to June 2015. Eurosurveillance.

[86] Chernet A., Neumayr A., Hatz C., Kling K., Sydow V., Rentsch K., Utzinger J., Probst-Hensch N., Marti H., Nickel B. (2018). Spectrum of infectious diseases among newly arrived Eritrean refugees in Switzerland: A cross-sectional study. Int. J. Public Health.

[87] Del Pinto R., Pietropaoli D., Russomando U., Evangelista P., Ferri C. (2018). Health status of Afro-Asian refugees in an Italian urban area: A cross-sectional monocentric study. Public Health.

[88] Jablonka A., Solbach P., Wöbse M., Manns M.P., Schmidt R.E., Wedemeyer H., Cornberg M., Behrens G.M.N., Hardtke S. (2017). Seroprevalence of antibodies and antigens against hepatitis A-E viruses in refugees and asylum seekers in Germany in 2015. Eur. J. Gastroenterol. Hepatol..

[89] Kortas A.Z., Polenz J., von Hayek J., Rüdiger S., Rottbauer W., Storr U., Wibmer T. (2017). Screening for infectious diseases among asylum seekers newly arrived in Germany in 2015: A systematic single-centre analysis. Public Health.

[90] Kurtuluş Ş., Sak Z.H.A., Can R. (2018). Chest Diseases in Refugees Living in a Tent Camp and in Turkish Citizens Living in the District: Ceylanpınar Experience. Turk. Thorac. J..

[91] Stevens K., Palmo T., Wangchuk T., Solomon S., Dierberg K., Hoffmann C.J. (2016). Hepatitis B prevalence and treatment needs among Tibetan refugees residing in India. J. Med. Virol..

[92] Turktan M., Ak O., Erdem H., Ozcengiz D., Hargreaves S., Kaya S., Karakoc E., Ozkan-Kuscu O., Tuncer-Ertem G., Tekin R. (2017). Community acquired infections among refugees leading to Intensive Care Unit admissions in Turkey. Int. J. Infect. Dis..

[93] Weinrich J.M., Diel R., Sauer M., Henes F.O., Meywald-Walter K., Adam G., Schön G., Bannas P. (2017). Yield of chest X-ray tuberculosis screening of immigrants during the European refugee crisis of 2015: A single-centre experience. Eur. Radiol..

[94] Masters P.J., Lanfranco P.J., Sneath E., Wade A.J., Huffam S., Pollard J., Standish J., McCloskey K., Athan E., O’Brien D.P. (2018). Health issues of refugees attending an infectious disease refugee health clinic in a regional Australian hospital. Aust. J. Gen. Pract..

[95] Serre-Delcor N., Ascaso C., Soriano-Arandes A., Collazos-Sanchez F., Treviño-Maruri B., Sulleiro E., Pou-Ciruelo D., Bocanegra-Garcia C., Molina-Romero I. (2018). Health Status of Asylum Seekers, Spain. Am. J. Trop. Med. Hyg..

[96] Warrington P., Tyrrell G., Choy K., Eisenbeis L., Long R., Cooper R. (2018). Prevalence of latent tuberculosis infection in Syrian refugees to Canada. Can. J. Public Health.

[97] Bozorgmehr K., Razum O., Saure D., Joggerst B., Szecsenyi J., Stock C. (2017). Yield of active screening for tuberculosis among asylum seekers in Germany: A systematic review and meta-analysis. Eurosurveillance.

[98] Arshad S., Bavan L., Gajari K., Paget S.N.J., Baussano I. (2010). Active screening at entry for tuberculosis among new immigrants: A systematic review and meta-analysis. Eur. Respir. J..

[99] Greenaway C., Thu Ma A., Kloda L.A., Klein M., Cnossen S., Schwarzer G., Shrier I. (2015). The Seroprevalence of Hepatitis C Antibodies in Immigrants and Refugees from Intermediate and High Endemic Countries: A Systematic Review and Meta-Analysis. PLoS ONE.

[100] Padovese V., Egidi A.M., Melillo Fenech T., Podda Connor M., Didero D., Costanzo G., Mirisola C. (2014). Migration and determinants of health: Clinical epidemiological characteristics of migrants in Malta (2010-11). J. Public Health.

[101] Eiset A.H., Wejse C. (2017). Review of infectious diseases in refugees and asylum seekers-current status and going forward. Public Health Rev..

[102] Isenring E., Fehr J., Gültekin N., Schlagenhauf P. (2018). Infectious disease profiles of Syrian and Eritrean migrants presenting in Europe: A systematic review. Travel. Med. Infect. Dis..

[103] de Smalen A.W., Ghorab H., Abd El Ghany M., Hill-Cawthorne G.A. (2017). Refugees and antimicrobial resistance: A systematic review. Travel. Med. Infect. Dis..

[104] Prestileo T., Pipitone G., Sanfilippo A., Ficalora A., Natoli G., Corrao S., Immigrant Take Care Advocacy Team (2021). Tuberculosis among Migrant Populations in Sicily: A Field Report. J. Trop. Med..

[105] Europe W. Report on the Health of Refugees and Migrants in the WHO European Region: No Public Health without Refugee and Migrant Health. https://www.euro.who.int/en/publications/html/report-on-the-health-of-refugees-and-migrants-in-the-who-european-region-no-public-health-without-refugee-and-migrant-health-2018/en/index.html.

[106] Cdcgov (2022). Diagnosing Latent TB Infection & TB Disease|TB|CDC. https://www.cdc.gov/tb/topic/testing/diagnosingltbi.htm.

[107] Rossi C., Shrier I., Marshall L., Cnossen S., Schwartzman K., Klein M.B., Schwarzer G., Greenaway C. (2012). Seroprevalence of chronic hepatitis B virus infection and prior immunity in immigrants and refugees: A systematic review and meta-analysis. PLoS ONE.

[108] Benson J., Donohue W. (2007). Hepatitis in refugees who settle in Australia. Aust. Fam. Physician.

[109] Sievert W., Altraif I., Razavi H.A., Abdo A., Ahmed E.A., Alomair A., Amarapurkar D., Chen C.H., Dou X., El Khayat H. (2011). A systematic review of hepatitis C virus epidemiology in Asia, Australia and Egypt. Liver Int..

[110] Madhava V., Burgess C., Drucker E. (2002). Epidemiology of chronic hepatitis C virus infection in sub-Saharan Africa. Lancet Infect. Dis..

[111] (2023). Global HIV & AIDS Statistics—Fact Sheet. https://www.unaids.org/en/resources/fact-sheet.

[112] Spiegel P.B., Bennedsen A.R., Claass J., Bruns L., Patterson N., Yiweza D., Schilperoord M. (2007). Prevalence of HIV infection in conflict-affected and displaced people in seven sub-Saharan African countries: A systematic review. Lancet.

[113] Baggaley R.F., Zenner D., Bird P., Hargreaves S., Griffiths C., Noori T., Friedland J.S., Nellums L.B., Pareek M. (2022). Prevention and treatment of infectious diseases in migrants in Europe in the era of universal health coverage. Lancet Public Health.

[114] Barnett E.D., Walker P.F. (2008). Role of immigrants and migrants in emerging infectious diseases. Med. Clin. N. Am..

[115] Gushulak B.D., MacPherson D.W. (2004). Globalization of Infectious Diseases: The Impact of Migration. Clin. Infect. Dis..

[116] Proença R., Mattos Souza F., Lisboa Bastos M., Caetano R., Braga J.U., Faerstein E., Trajman A. (2020). Active and latent tuberculosis in refugees and asylum seekers: A systematic review and meta-analysis. BMC Public Health.

[117] Hönekopp J., Linden A.H. (2022). Heterogeneity estimates in a biased world. PLoS ONE.

