# Supplementary material for: Communicable Diseases Prevalence among Refugees and Asylum Seekers: Systematic Review and Meta-Analysis
Source: Infect Dis Rep. 2023 Mar 31;15(2):188–203. doi: 10.3390/idr15020020 (PMC10138615; doi:10.3390/idr15020020)
Supplement: Supplementary file 1 [file idr-15-00020-s001.zip › idr-2224175-supplementary.pdf]

Supplementary Materials

S 1: PubMed search strategy history

(((((((((((((((((((((((((Pathogen Transmission[Title/Abstract]) OR Transmission, Pathogen[Title/Abstract]) OR Infectious Disease Transmission[Title/Abstract]) OR Transmission, Infectious Disease[Title/Abstract]) OR Transmission of Infectious Disease[Title/Abstract]) OR Infection Transmission[Title/Abstract]) OR Transmission, Infection[Title/Abstract]) OR Communicable Disease Transmission[Title/Abstract]) OR Disease Transmission, Communicable[Title/Abstract]) OR Transmission, Communicable Disease[Title/Abstract]) OR Autochthonous Transmission[Title/Abstract]) OR Autochthonous Transmissions[Title/Abstract]) OR Transmission, Autochthonous[Title/Abstract]) OR Transmissions, Autochthonous[Title/Abstract]) OR Infectious Disease Transmission, Horizontal[Title/Abstract]) OR Pathogen Transmission, Horizontal[Title/Abstract]) OR Horizontal Transmission of Infectious Disease[Title/Abstract]) OR Horizontal Transmission of Infection[Title/Abstract]) OR Infection Horizontal Transmission[Title/Abstract]) OR Infection Transmission, Horizontal[Title/Abstract])) OR Disease Transmission, Infectious[MeSH Terms])) OR ((Communicable Diseases, Emerging[MeSH Terms]) OR ((((((((((((((((((((((((((((((((((((Communicable Disease, Emerging[Title/Abstract]) OR Disease, Emerging Communicable[Title/Abstract]) OR Diseases, Emerging Communicable[Title/Abstract]) OR Emerging Communicable Disease[Title/Abstract]) OR Emerging Communicable Diseases[Title/Abstract]) OR Infectious Diseases, Emerging[Title/Abstract]) OR Disease, Emerging Infectious[Title/Abstract]) OR Diseases, Emerging Infectious[Title/Abstract]) OR Emerging Infectious Disease[Title/Abstract]) OR Emerging Infectious Diseases[Title/Abstract]) OR Infectious Disease, Emerging[Title/Abstract]) OR Communicable Diseases, Reemerging[Title/Abstract]) OR Communicable Disease, Reemerging[Title/Abstract]) OR Disease, Reemerging Communicable[Title/Abstract]) OR Diseases, Reemerging Communicable[Title/Abstract]) OR Reemerging Communicable Disease[Title/Abstract]) OR Reemerging Communicable Diseases[Title/Abstract]) OR Infectious Diseases, Re-Emerging[Title/Abstract]) OR Disease, Re-Emerging Infectious[Title/Abstract]) OR Diseases, Re-Emerging Infectious[Title/Abstract]) OR Infectious Disease, Re-Emerging[Title/Abstract]) OR Infectious Diseases, Re Emerging[Title/Abstract]) OR Re-Emerging Infectious Disease[Title/Abstract]) OR Re- Emerging Infectious Diseases[Title/Abstract]) OR Infectious Diseases, Reemerging[Title/Abstract]) OR Disease, Reemerging Infectious[Title/Abstract]) OR Diseases, Reemerging Infectious[Title/Abstract]) OR Infectious Disease, Reemerging[Title/Abstract]) OR Reemerging Infectious Disease[Title/Abstract]) OR Reemerging Infectious Diseases[Title/Abstract]) OR Communicable Diseases, Re-Emerging[Title/Abstract]) OR Communicable Disease, Re-Emerging[Title/Abstract]) OR Communicable Diseases, Re Emerging[Title/Abstract]) OR Disease, Re-Emerging Communicable[Title/Abstract]) OR Diseases, Re-Emerging Communicable[Title/Abstract]) OR Re-Emerging Communicable Disease[Title/Abstract]) OR Re-Emerging Communicable Diseases[Title/Abstract])))) OR ((((((((Infectious Disease[Title/Abstract]) OR Diseases, Infectious[Title/Abstract]) OR Disease, Infectious[Title/Abstract]) OR Infectious Diseases[Title/Abstract]) OR Diseases, Communicable[Title/Abstract]) OR Disease, Communicable[Title/Abstract]) OR Communicable Disease[Title/Abstract])) OR Communicable diseases[MeSH Terms])) AND ((((((((((Refugee Camp[Title/Abstract]) OR Camps, Refugee[Title/Abstract]) OR Camps, Refugee[Title/Abstract]) OR Camp, Refugee[Title/Abstract]) OR Refugee Camps[Title/Abstract]) OR Seekers, Asylum[Title/Abstract]) OR Asylum Seeker[Title/Abstract]) OR Asylum Seekers[Title/Abstract]) OR Refugee[Title/Abstract]) OR refugee[MeSH Terms]

S 2: Scopus search strategy history

(TITLE-ABS-KEY (communicable AND disease) OR TITLE-ABS-KEY (disease, AND communicable) OR TITLE-ABS-KEY (diseases, AND communicable) OR TITLE-ABS-KEY (infectious AND diseases) OR TITLE-ABS-KEY (disease, AND infectious) OR TITLE-ABS-KEY (diseases, AND infectious) OR TITLE-ABS-KEY (infectious AND disease) OR TITLE-ABS-KEY (communicable AND disease, AND emerging) OR TITLE-ABS-KEY (disease, AND emerging AND communicable) OR TITLE-ABS-KEY (diseases, AND emerging AND communicable) OR TITLE-ABS-KEY (emerging AND communicable AND disease) OR TITLE-ABS-KEY (emerging AND communicable AND diseases) OR TITLE-ABS-KEY (infectious AND diseases, AND emerging) OR TITLE-ABS-KEY (disease, AND emerging AND infectious) OR TITLE-ABS-KEY (diseases, AND emerging AND infectious) OR TITLE-ABS-KEY (emerging AND infectious AND disease) OR TITLE-ABS-KEY (emerging AND infectious AND diseases) OR TITLE- ABS-KEY (infectious AND disease, AND emerging) OR TITLE-ABS-KEY (communicable AND diseases, AND reemerging) OR TITLE-ABS-KEY (communicable AND disease, AND reemerging) OR TITLE- ABS-KEY (disease, AND reemerging AND communicable) OR TITLE-ABS-KEY (diseases, AND reemerging AND communicable) OR TITLE-ABS-KEY (reemerging AND communicable AND disease) OR TITLE-ABS-KEY (reemerging AND communicable AND diseases)) OR (TITLE-ABS-KEY (infectious AND diseases, AND re-emerging) OR TITLE-ABS-KEY (disease, AND re-emerging AND infectious) OR TITLE-ABS-KEY (diseases, AND re-emerging AND infectious) OR TITLE-ABS-KEY (infectious AND disease, AND re-emerging) OR TITLE-ABS-KEY (infectious AND diseases, AND re AND emerging) OR TITLE-ABS-KEY (re-emerging AND infectious AND disease) OR TITLE-ABS-KEY (re-emerging AND infectious AND diseases) OR TITLE-ABS-KEY (infectious AND diseases, AND reemerging) OR TITLE-ABS-KEY (disease, AND reemerging AND infectious) OR TITLE-ABS-KEY (diseases, AND reemerging AND infectious) OR TITLE-ABS-KEY (infectious AND disease, AND reemerging) OR TITLE-ABS-KEY (reemerging AND infectious AND disease) OR TITLE-ABS-KEY (reemerging AND infectious AND diseases) OR TITLE-ABS-KEY (communicable AND diseases, AND re-emerging) OR TITLE-ABS-KEY (communicable AND disease, AND re-emerging) OR TITLE-ABS-KEY (communicable AND diseases, AND re AND emerging) OR TITLE-ABS-KEY (disease, AND re-emerging AND communicable) OR TITLE-ABS-KEY (diseases, AND re-emerging AND communicable) OR TITLE- ABS-KEY (re-emerging AND communicable AND disease) OR TITLE-ABS-KEY (re-emerging AND communicable AND diseases) OR TITLE-ABS-KEY (pathogen AND transmission) OR TITLE-ABS-KEY (transmission, AND pathogen) OR TITLE-ABS-KEY (infectious AND disease AND transmission) OR TITLE-ABS-KEY (transmission, AND infectious AND disease)) OR (TITLE-ABS-KEY (transmission AND of AND infectious AND disease) OR TITLE-ABS-KEY (infection AND transmission) OR TITLE- ABS-KEY (transmission, AND infection) OR TITLE-ABS-KEY (communicable AND disease AND transmission) OR TITLE-ABS-KEY (disease AND transmission, AND communicable) OR TITLE-ABS- KEY (transmission, AND communicable AND disease) OR TITLE-ABS-KEY (autochthonous AND transmission) OR TITLE-ABS-KEY (autochthonous AND transmissions) OR TITLE-ABS-KEY (transmission, AND autochthonous) OR TITLE-ABS-KEY (transmissions, AND autochthonous) OR TITLE-ABS-KEY (infectious AND disease AND transmission, AND horizontal) OR TITLE-ABS-KEY (pathogen AND transmission, AND horizontal) OR TITLE-ABS-KEY (horizontal AND transmission AND of AND infectious AND disease) OR TITLE-ABS-KEY (horizontal AND transmission AND of AND infection) OR TITLE-ABS-KEY (infection AND horizontal AND transmission) OR TITLE-ABS-KEY (infection AND transmission, AND horizontal)) OR (TITLE-ABS-KEY (tuberculosis) OR TITLE-ABS-KEY (tuberculosis)) OR (TITLE-ABS-KEY (hepatitis) OR TITLE-ABS-KEY (hepatitis)) OR (TITLE-ABS- KEY (hiv) OR TITLE-ABS-KEY (human AND immunodeficiency AND virus) OR TITLE-ABS-KEY (immunodeficiency AND virus, AND human) OR TITLE-ABS-KEY (immunodeficiency AND viruses, AND human) OR TITLE-ABS-KEY (virus, AND human AND immunodeficiency) OR TITLE-ABS-KEY (viruses, AND human AND immunodeficiency) OR TITLE-ABS-KEY (human AND immunodeficiency AND viruses) OR TITLE-ABS-KEY (human AND t AND cell AND lymphotropic AND virus AND type AND iii) OR TITLE-ABS-KEY (human AND t-cell AND lymphotropic AND virus AND type AND iii) OR TITLE-ABS-KEY (lav-htlv-iii) OR TITLE-ABS-KEY (lymphadenopathy-associated AND virus) OR TITLE-ABS-KEY (lymphadenopathy AND associated AND virus) OR TITLE-ABS-KEY (lymphadenopathy-associated AND viruses) OR TITLE-ABS-KEY (virus, AND lymphadenopathy-associated) OR TITLE-ABS-KEY (viruses, AND lymphadenopathy-associated) OR TITLE-ABS-KEY (human AND t AND lymphotropic AND virus AND type AND iii) OR TITLE-ABS-KEY (human

AND t-lymphotropic AND virus AND type AND iii) OR TITLE-ABS-KEY (aids AND virus) OR TITLE-ABS-KEY (aids AND viruses) OR TITLE-ABS-KEY (virus, AND aids) OR TITLE-ABS-KEY (viruses, AND aids) OR TITLE-ABS-KEY (acquired AND immune AND deficiency AND syndrome AND virus) OR TITLE- ABS-KEY (acquired AND immunodeficiency AND syndrome AND virus) OR TITLE-ABS-KEY (htlv-iii)) OR (TITLE-ABS-KEY (hiv) OR TITLE-ABS-KEY (hiv)) OR (TITLE-ABS-KEY (syphilis) OR TITLE-ABS-KEY (syphilis)) OR (TITLE-ABS-KEY (gonorrhea) OR TITLE-ABS-KEY (gonorrhea)) OR (TITLE-ABS-KEY (chlamydia) OR TITLE-ABS-KEY (chlamydia)) OR (TITLE-ABS-KEY (chlamydia AND infections) OR TITLE-ABS-KEY (chlamydia)) OR (TITLE-ABS-KEY (sexutitle-abs- keyy AND transmitted AND diseases) OR TITLE-ABS-KEY (disease, AND sexutitle-abs-keyy AND transmitted) OR TITLE-ABS-KEY (sexutitle-abs-keyy AND transmitted AND disease) OR TITLE-ABS- KEY (stis) OR TITLE-ABS-KEY (sti) OR TITLE-ABS-KEY (venereal AND diseases) OR TITLE-ABS-KEY (disease, AND venereal) OR TITLE-ABS-KEY (diseases, AND venereal) OR TITLE-ABS-KEY (venereal AND disease) OR TITLE-ABS-KEY (sexutitle-abs-keyy AND transmitted AND infections) OR TITLE-ABS-KEY (infection, AND sexutitle-abs-keyy AND transmitted) OR TITLE-ABS-KEY (infections, AND sexutitle-abs-keyy AND transmitted) OR TITLE-ABS-KEY (sexutitle-abs-keyy AND transmitted AND infection) OR TITLE-ABS-KEY (transmitted AND infection, AND sexutitle-abs-keyy) OR TITLE- ABS-KEY (transmitted AND infections, AND sexutitle-abs-keyy) OR TITLE-ABS-KEY (stds)) OR (TITLE-ABS-KEY (sexutitle-abs-keyy AND transmitted AND disease) OR TITLE-ABS-KEY (sexutitle- abs-keyy AND transmitted AND disease)) OR (TITLE-ABS-KEY (parasitic AND diseases) OR TITLE- ABS-KEY (disease, AND parasitic) OR TITLE-ABS-KEY (diseases, AND parasitic) OR TITLE-ABS- KEY (parasitic AND disease) OR TITLE-ABS-KEY (parasitic AND infections) OR TITLE-ABS-KEY (infection, AND parasitic) OR TITLE-ABS-KEY (infections, AND parasitic) OR TITLE-ABS-KEY (parasitic AND infection) OR TITLE-ABS-KEY (parasite AND infections) OR TITLE-ABS-KEY (infection, AND parasite) OR TITLE-ABS-KEY (infections, AND parasite) OR TITLE-ABS-KEY (parasite AND infection) OR TITLE-ABS-KEY (malaria) OR TITLE-ABS-KEY (remittent AND fever) OR TITLE-ABS-KEY (fever, AND remittent) OR TITLE-ABS-KEY (paludism) OR TITLE-ABS-KEY (plasmodium AND infections) OR TITLE-ABS-KEY (infections, AND plasmodium) OR TITLE-ABS-KEY (infection, AND plasmodium) OR TITLE-ABS-KEY (plasmodium AND infection) OR TITLE-ABS-KEY (marsh AND fever) OR TITLE-ABS-KEY (fever, AND marsh)) AND (TITLE-ABS-KEY (refugee) OR TITLE-ABS-KEY (asylum AND seekers) OR TITLE-ABS-KEY (asylum AND seeker) OR TITLE-ABS-KEY (seekers,asylum) OR TITLE-ABS-KEY (refugee AND camps) OR TITLE-ABS-KEY (camp,refugee) OR TITLE-ABS-KEY (camps,refugee) OR TITLE-ABS-KEY (refugee AND camp))

S 3: Embase search strategy history

'communicable disease'/exp OR 'communicable disease' OR 'communicable diseases':ab,ti OR 'communicable diseases, emerging':ab,ti OR 'contagious disease':ab,ti OR 'contagious infection':ab,ti OR 'disease, communicable':ab,ti OR 'transmissible disease':ab,ti OR 'transmissible infection':ab,ti OR 'tuberculosis'/exp OR 'tuberculosis' OR 'tuberculosis':ab,ti OR 'active tuberculosis':ab,ti OR 'chronic tuberculosis':ab,ti OR 'minimum tuberculosis':ab,ti OR 'mycobacterium tuberculosis infection':ab,ti OR 'tuberculosis, cardiovascular':ab,ti OR 'tuberculosis, endocrine':ab,ti OR 'tuberculous infection':ab,ti OR 'tuberculous lesion':ab,ti OR 'minimal tuberculosis':ab,ti OR 'hepatitis'/exp OR 'hepatitis' OR 'hepatitis':ab,ti OR 'fulminant hepatitis':ab,ti OR 'liver infection':ab,ti OR 'liver inflammation':ab,ti OR 'liver inflammatory disease':ab,ti OR 'subacute hepatitis':ab,ti OR 'human immunodeficiency virus'/exp OR 'human immunodeficiency virus' OR 'human immunodeficiency virus 1':ab,ti OR 'human immunodeficiency virus 2':ab,ti OR 'human immunodeficiency virus':ab,ti OR 'sexually transmitted disease'/exp OR 'sexually transmitted disease' OR 'sexually transmitted disease':ab,ti OR 'gonorrhea':ab,ti OR 'syphilis':ab,ti OR 'sexual disease transmission':ab,ti OR 'sexually transmitted diseases':ab,ti OR 'sexually transmitted diseases, bacterial':ab,ti OR 'sexually transmitted diseases, viral':ab,ti OR 'sexually transmitted infection':ab,ti OR 'std':ab,ti OR 'vd':ab,ti OR 'veneral disease':ab,ti OR 'venereal disease':ab,ti OR 'venereal infection':ab,ti OR 'parasitosis'/exp OR 'parasitosis' OR 'parasitosis':ab,ti OR 'malaria'/exp OR 'malaria' OR 'malaria':ab,ti OR 'malaria infection':ab,ti OR 'malaria transmission':ab,ti OR 'paludism':ab,ti OR 'swamp fever':ab,ti AND 'refugee'/exp OR 'refugee':ab,ti OR 'asylum seeker':ab,ti

S 4: Cinahl search strategy history

TI ((((MH "Communicable Diseases") OR "COMMUNICABLE disease" OR TX Communicable Diseases OR TI Communicable Diseases OR AB Communicable Diseases OR MW Communicable Diseases OR MH Communicable Diseases OR MJ Communicable Diseases OR MM Communicable Diseases OR SU Communicable Diseases) OR (TX Infectious Disease OR TI Infectious Disease OR AB Infectious Disease OR MW Infectious Disease OR MH Infectious Disease OR MJ Infectious Disease OR MM Infectious Disease OR SU Infectious Disease OR (MH "Communicable Diseases") OR "infectious disease")) OR (TX Tuberculosis OR TI Tuberculosis OR AB Tuberculosis OR MW Tuberculosis OR MH Tuberculosis OR MJ Tuberculosis OR MM Tuberculosis OR SU Tuberculosis OR (MH "Tuberculosis") OR "tuberculosis") OR (TX Hepatitis OR TI Hepatitis OR AB Hepatitis OR MW Hepatitis OR MH Hepatitis OR MJ Hepatitis OR MM Hepatitis OR SU Hepatitis OR (MM "Hepatitis+") OR "HEPATITIS" OR (MM "Hepatitis, Viral, Human+") OR (MM "Hepatitis, Chronic+") OR (MM "Hepatitis Viruses") OR (MM "Hepatitis C, Chronic") OR (MM "Hepatitis B, Chronic") OR (MM "Hepatitis D") OR (MM "Hepatitis C+") OR (MM "Hepatitis A")) OR (TX HIV OR TI HIV OR AB HIV OR MW HIV OR MH HIV OR MJ HIV OR MM HIV OR SU HIV OR "hiv" OR (MM "HIV Infections+") OR(MM "HIV-Infected Patients+") OR (MM "Human Immunodeficiency Virus+") OR "Human Immunodeficiency Virus" OR "HIV" OR (MM "HIV Infections+") OR (MM "HIV-Infected Patients+") OR (MM "Human Immunodeficiency Virus+") OR "Human Immunodeficiency Virus") OR ((MM "Sexually Transmitted Diseases, Viral+") OR (MM "Sexually Transmitted Diseases, Protozoal+") OR (MM "Sexually Transmitted Diseases, Fungal+") OR (MM "Sexually Transmitted Diseases, Bacterial+") OR (MM "Sexually Transmitted Diseases+") OR "SEXUALLY TRANSMITTED DISEASE" OR TX sexually transmitted infections OR TI sexually transmitted infections OR AB sexually transmitted infections OR MW sexually transmitted infections OR MH sexually transmitted infections OR MJ sexually transmitted infections OR MM sexually transmitted infections OR SU sexually transmitted infections) OR (TX Syphilis OR TI Syphilis OR AB Syphilis OR MW Syphilis OR MH Syphilis OR MJ Syphilis OR MM Syphilis OR SU Syphilis OR (MM "Syphilis") OR "syphilis") OR (TX Gonorrhea OR TI Gonorrhea OR AB Gonorrhea OR MW Gonorrhea OR MH Gonorrhea OR MJ Gonorrhea OR MM Gonorrhea OR SU Gonorrhea OR (MM "Gonorrhea+") OR "GONORRHEA" OR (MM "Neisseria")) OR (TX Chlamydia OR TI Chlamydia OR AB Chlamydia OR MW Chlamydia OR MH Chlamydia OR MJ Chlamydia OR MM Chlamydia OR SU Chlamydia OR (MM "Chlamydia+") OR "CHLAMYDIA" OR (MM "Chlamydia Infections+") OR (MM "Chlamydia Trachomatis")) OR ((MM "Central Nervous System Parasitic Infections+") OR (MM "Coinfection") OR (MM "Eye Infections, Parasitic+") OR (MM "Helminthiasis+") OR (MM "Liver Diseases, Parasitic+") OR (MM "Intestinal Diseases, Parasitic+") OR (MM "Lung Diseases, Parasitic") OR (MM "Opportunistic Infections+") OR (MH "Parasitemia") OR (MM "Pregnancy Complications, Parasitic") OR (MM "Protozoan Infections+") OR (MM "Skin Diseases, Parasitic+") OR (MM "Parasitic Diseases+") OR TX Parasitic Diseases OR TI Parasitic Diseases OR AB Parasitic Diseases OR MW Parasitic Diseases OR MH Parasitic Diseases OR MJ Parasitic Diseases OR MM Parasitic Diseases OR SU Parasitic Diseases)) AND TI (TX REFUGEE OR TI refugee OR AB refugee OR MH refugee OR MM refugee OR TX asylum seeker OR TI asylum seeker OR AB asylum seeker OR MH asylum seeker OR MM asylum seeker)

S 5: Systematic review data extraction sheet

**Study Number**	**Study 1**	**Study 2**	**…**
Notes			
General study Purpose (Aim)			
Risk of Bias (Of the Applicable) (0-3)/ 10 =Low Risk<br>(4-7)/10 = Moderate Risk (8-10)/10= High risk			

Hoy Et Al Quality 1 Yes=Low Risk= 0 No=High Risk=1
Hoy Et Al Quality 2
Hoy Et Al Quality 3
Hoy Et Al Quality 4
Internal Validity
Score/4
External Validity
Score/6
Hoy Et Al Quality 5
Hoy Et Al Quality 6
Hoy Et Al Quality 7
Hoy Et Al Quality 8
Hoy Et Al Quality 9
Hoy Et Al Quality 10
Include?
Year of Publication
Year of Study *Time of the study
Final Year Of Study
First Author
Country
population
Country of Origin
Sampling Method
Sample Coverage
Instrument
Study Type (Design)
General Disease Under Observation
Disease Under Observation
Type of TB Study
Sample Size
Denominator/ Numerator Found
Prevalence
TB Prevalence
HBV Prevalence
HIV Prevalence

S 6: Risk of Bias Tool, Hoy et al, 2012

Name of author(s): Year of publication:

Name of paper/study:-

This tool is designed to assess the risk of bias in population-based prevalence studies. Please read the additional notes for each item when initially using the tool. Note: If there is insufficient information in the article to permit a judgement for a particular item, please answer No (HIGH RISK) for that particular item.

Risk of bias item	Criteria for answers (please circle one option)	Additional notes and examples
External Validity		
1. Was the study"s target population a close representation of the national population in relation to relevant variables, e.g. age, sex, occupation?	• Yes (LOW RISK): The study"s target population was a close representation of the national population.<br>• No (HIGH RISK): The study"s target population was clearly NOT representative of the national population.	The target population refers to the group of people or entities to which the results of the study will be generalised. Examples:<br>• The study was a national health survey of people 15 years and over and the sample was drawn from a list that included all individuals in the population aged 15 years and over. The answer is: Yes (LOW RISK).<br>• The study was conducted in one province only, and it is not clear if this was representative of the national population. The answer is: No (HIGH RISK).<br>• The study was undertaken in one village only and it is clear this was not representative of the national population. The answer is:<br>No (HIGH RISK).
2. Was the sampling frame a true or close representation of the target population?	• Yes (LOW RISK): The sampling frame was a true or close representation of the target population.<br>• No (HIGH RISK): The sampling frame was NOT a true or close representation of the target population.	The sampling frame is a list of the sampling units in the target population and the study sample is drawn from this list. Examples:<br>• The sampling frame was a list of almost every individual within the target population. The answer is: Yes (LOW RISK).<br>• The cluster sampling method was used and the sample of clusters/villages was drawn from a list of all villages in the target population. The answer is: Yes (LOW RISK).<br>• The sampling frame was a list of just one particular ethnic group

		within the overall target population, which comprised many groups. The answer is: No (HIGH RISK).
3. Was some form of random selection used to select the sample, OR, was a census undertaken?	• Yes (LOW RISK): A census was undertaken, OR, some form of random selection was used to select the sample (e.g. simple random sampling, stratified random sampling, cluster sampling, systematic sampling).<br><br>• No (HIGH RISK): A census was NOT undertaken, AND some form of random selection was NOT used to select the sample.	A census collects information from every unit in the sampling frame. In a survey, only part of the sampling frame is sampled. In these instances, random selection of the sample helps minimise study bias. Examples:<br><br>• The sample was selected using simple random sampling. The answer is: Yes (LOW RISK).<br><br>• The target population was the village and every person in the village was sampled. The answer is: Yes (LOW RISK).<br><br>• The nearest villages to the capital city were selected in order to save on the cost of fuel. The answer is: No (HIGH RISK).
4. Was the likelihood of non-response bias minimal?	• Yes (LOW RISK): The response rate for the study was >/=75%, OR, an analysis was performed that showed no significant difference in relevant demographic characteristics between responders and non-responders<br><br>• No (HIGH RISK): The response rate was <75%, and if any analysis comparing responders and non-responders was done, it showed a significant difference in relevant demographic characteristics between responders and non-responders.	Examples:<br><br>• The response rate was 68%; however, the researchers did an analysis and found no significant difference between responders and non-responders in terms of age, sex, occupation and socio- economic status. The answer is: Yes (LOW RISK).<br><br>• The response rate was 65% and the researchers did NOT carry out an analysis to compare relevant demographic characteristics between responders and non-responders. The answer is: No (HIGH RISK).<br><br>• The response rate was 69% and the researchers did an analysis and found a significant difference in age, sex and socio-economic status between responders and non-responders. The answer is: No (HIGH RISK).

Internal Validity

5. Were data collected directly from the subjects (as opposed to a proxy)?	• Yes (LOW RISK): All data were collected directly from the subjects.<br><br>• No (HIGH RISK): In some instances, data were collected from a proxy.	A proxy is a representative of the subject. Examples:<br><br>• All eligible subjects in the household were interviewed separately. The answer is: Yes (LOW RISK).<br><br>• A representative of the household was interviewed and questioned about the presence of low back pain in each household member. The answer is: No (HIGH RISK).
6. Was an acceptable case definition used in the study?	• Yes (LOW RISK): An acceptable case definition was used.<br><br>• No (HIGH RISK): An acceptable case definition was NOT used.	• For a study on low back pain, the following case definition was used: "Low back pain is defined as activity-limiting pain lasting more than one day in the area on the posterior aspect of the body from the bottom of the 12th rib to the lower gluteal folds." The answer is: Yes (LOW RISK).<br><br>• For a study on back pain, there was no description of the specific anatomical location „back" referred to. The answer is: No (HIGH RISK).<br><br>• For a study on osteoarthritis, the following case definition was used: "Symptomatic osteoarthritis of the hip or knee, radiologically confirmed as Kellgren-Lawrence grade 2-4". The answer is: LOW RISK.
7. Was the study instrument that measured the parameter of interest (e.g. prevalence of low back pain) shown to have reliability and validity (if necessary)?	• Yes (LOW RISK): The study instrument had been shown to have reliability and validity (if this was necessary), e.g. test-re- test, piloting, validation in a previous study, etc.<br><br>• No (HIGH RISK): The study instrument had NOT been shown to have reliability or validity (if this was necessary).	• The authors used the COPCORD questionnaire, which had previously been validated. They also tested the inter-rater reliability of the questionnaire. The answer is: Yes (LOW RISK).<br><br>• The authors developed their own questionnaire and did not test this for validity or reliability. The answer is: No (HIGH RISK).
8. Was the same mode of data collection used for all subjects?	• Yes (LOW RISK): The same mode of data collection was used for all subjects.<br><br>• No (HIGH RISK): The same mode of data collection was NOT used for all subjects.	The mode of data collection is the method used for collecting information from the subjects. The most common modes are face-to- face interviews, telephone interviews and self-administered questionnaires. Examples:<br><br>• All eligible subjects had a face-to-face interview. The answer is:<br>Yes (LOW RISK).

		• Some subjects were interviewed over the telephone and some filled in postal questionnaires. The answer is: No (HIGH RISK).
9. Was the length of the shortest prevalence period for the parameter of interest appropriate?	• Yes (LOW RISK): The shortest prevalence period for the parameter of interest was appropriate (e.g. point prevalence, one-week prevalence, one-year prevalence).<br><br>• No (HIGH RISK): The shortest prevalence period for the parameter of interest was not appropriate (e.g. lifetime prevalence)	The prevalence period is the period that the subject is asked about e.g. "Have you experienced low back pain over the previous year?" In this example, the prevalence period is one year. The longer the prevalence period, the greater the likelihood of the subject forgetting if they experienced the symptom of interest (e.g. low back pain). Examples:<br><br>• Subjects were asked about pain over the past week. The answer is:<br>Yes (LOW RISK).<br><br>• Subjects were only asked about pain over the past three years. The answer is: No (HIGH RISK).
10. Were the numerator(s) and denominato r(s) for the parameter of interest appropriate?	• Yes (LOW RISK): The paper presented appropriate numerator(s) AND denominator(s) for the parameter of interest (e.g. the prevalence of low back pain).<br><br>• No (HIGH RISK): The paper did present numerator(s) AND denominator(s) for the parameter of interest but one or more of these were inappropriate.	There may be errors in the calculation and/or reporting of the numerator and/or denominator. Examples:<br><br>• There were no errors in the reporting of the numerator(s) AND denominator(s) for the prevalence of low back pain. The answer is: Yes (LOW RISK).<br><br>• In reporting the overall prevalence of low back pain (in both men and women), the authors accidentally used the population of women as the denominator rather than the combined population. The answer is: No (HIGH RISK).
11. Summary item on the overall risk of study bias		
• LOW RISK OF BIAS: Further research is very unlikely to change our confidence in the estimate.<br><br>• MODERATE RISK OF BIAS: Further research is likely to have an important impact on our confidence in the estimate and may change the estimate.		

- **HIGH RISK OF BIAS:** Further research is very likely to have an important impact on our confidence in the estimate and is likely to change the estimate.

S 7: PRISMA 2009 Checklist

Section/topic	#	Checklist item	Reported on page #
TITLE			
Title	1	Identify the report as a systematic review, meta-analysis, or both.	
ABSTRACT			
Structured summary	2	Provide a structured summary including, as applicable: background; objectives; data sources; study eligibility criteria, participants, and interventions; study appraisal and synthesis methods; results; limitations; conclusions and implications of key findings; systematic review registration number.	
INTRODUCTION			
Rationale	3	Describe the rationale for the review in the context of what is already known.	
Objectives	4	Provide an explicit statement of questions being addressed with reference to participants, interventions, comparisons, outcomes, and study design (PICOS).	
METHODS			
Protocol and registration	5	Indicate if a review protocol exists, if and where it can be accessed (e.g., Web address), and, if available, provide registration information including registration number.	
Eligibility criteria	6	Specify study characteristics (e.g., PICOS, length of follow-up) and report characteristics (e.g., years considered, language, publication status) used as criteria for eligibility, giving rationale.	
Information sources	7	Describe all information sources (e.g., databases with dates of coverage, contact with study authors to identify additional studies) in the search and date last searched.	
Search	8	Present full electronic search strategy for at least one database, including any limits used, such that it could be repeated.	
Study selection	9	State the process for selecting studies (i.e., screening, eligibility, included in systematic review, and, if applicable, included in the meta-analysis).	
Data collection process	10	Describe method of data extraction from reports (e.g., piloted forms, independently, in duplicate) and any	

		processes for obtaining and confirming data from investigators.	
Data items	11	List and define all variables for which data were sought (e.g., PICOS, funding sources) and any assumptions and simplifications made.	
Risk of bias in individual studies	12	Describe methods used for assessing risk of bias of individual studies (including specification of whether this was done at the study or outcome level), and how this information is to be used in any data synthesis.	
Summary measures	13	State the principal summary measures (e.g., risk ratio, difference in means).	
Synthesis of results	14	Describe the methods of handling data and combining results of studies, if done, including measures of consistency (e.g., I2) for each meta-analysis.	

Page 1 of 2

**Section/topic**	#	**Checklist item**	**Reported on page #**
Risk of bias across studies	15	Specify any assessment of risk of bias that may affect the cumulative evidence (e.g., publication bias, selective reporting within studies).	
Additional analyses	16	Describe methods of additional analyses (e.g., sensitivity or subgroup analyses, meta-regression), if done, indicating which were pre-specified.	
RESULTS			
Study selection	17	Give numbers of studies screened, assessed for eligibility, and included in the review, with reasons for exclusions at each stage, ideally with a flow diagram.	
Study characteristics	18	For each study, present characteristics for which data were extracted (e.g., study size, PICOS, follow-up period) and provide the citations.	
Risk of bias within studies	19	Present data on risk of bias of each study and, if available, any outcome level assessment (see item 12).	
Results of individual studies	20	For all outcomes considered (benefits or harms), present, for each study: (a) simple summary data for each intervention group (b) effect estimates and confidence intervals, ideally with a forest plot.	
Synthesis of results	21	Present results of each meta-analysis done, including confidence intervals and measures of consistency.	
Risk of bias across studies	22	Present results of any assessment of risk of bias across studies (see Item 15).	

Additional analysis	23	Give results of additional analyses, if done (e.g., sensitivity or subgroup analyses, meta-regression [see Item 16]).
DISCUSSION		
Summary of evidence	24	Summarize the main findings including the strength of evidence for each main outcome; consider their relevance to key groups (e.g., healthcare providers, users, and policy makers).
Limitations	25	Discuss limitations at study and outcome level (e.g., risk of bias), and at review-level (e.g., incomplete retrieval of identified research, reporting bias).
Conclusions	26	Provide a general interpretation of the results in the context of other evidence, and implications for future research.
FUNDING		
Funding	27	Describe sources of funding for the systematic review and other support (e.g., supply of data); role of funders for the systematic review.

From: Moher D, Liberati A, Tetzlaff J, Altman DG, The PRISMA Group (2009). Preferred Reporting Items for Systematic Reviews and Meta-Analyses: The PRISMA Statement. PLoS Med 6(7): e1000097. doi:10.1371/journal.pmed1000097

S 8: WHO Estimates of TB incidence, HBV and HCV prevalence, and HIV-people living, based, 2022

WHO [a] Region	TB [b] Incidence rate Rates per 100 000 population	HBV [c] Prevalence	HCV [d] Prevalence	HIV [e] -People living
Africa	254	6.1	1.0	25,600,000
South East Asia	240	2.0	0.5	3,500,000
Global	140	3.5	1.0	36,700,000
Eastern Mediterranean	114	3.3	2.3	360,000
Western Pacific	95	6.2	0.7	1,500,000
Europe	32	1.6	1.5	2,400,000
The Americas	27	0.7	0.7	3,300,000

[a] world health organization; [b], tuberculosis; [c], hepatitis B virus; [d], hepatitis C virus; [e], human immunodeficiency virus

S 9: Forest plots of the meta-analysis by the disease

Plot 9.1: Tuberculosis forest plot by origin region

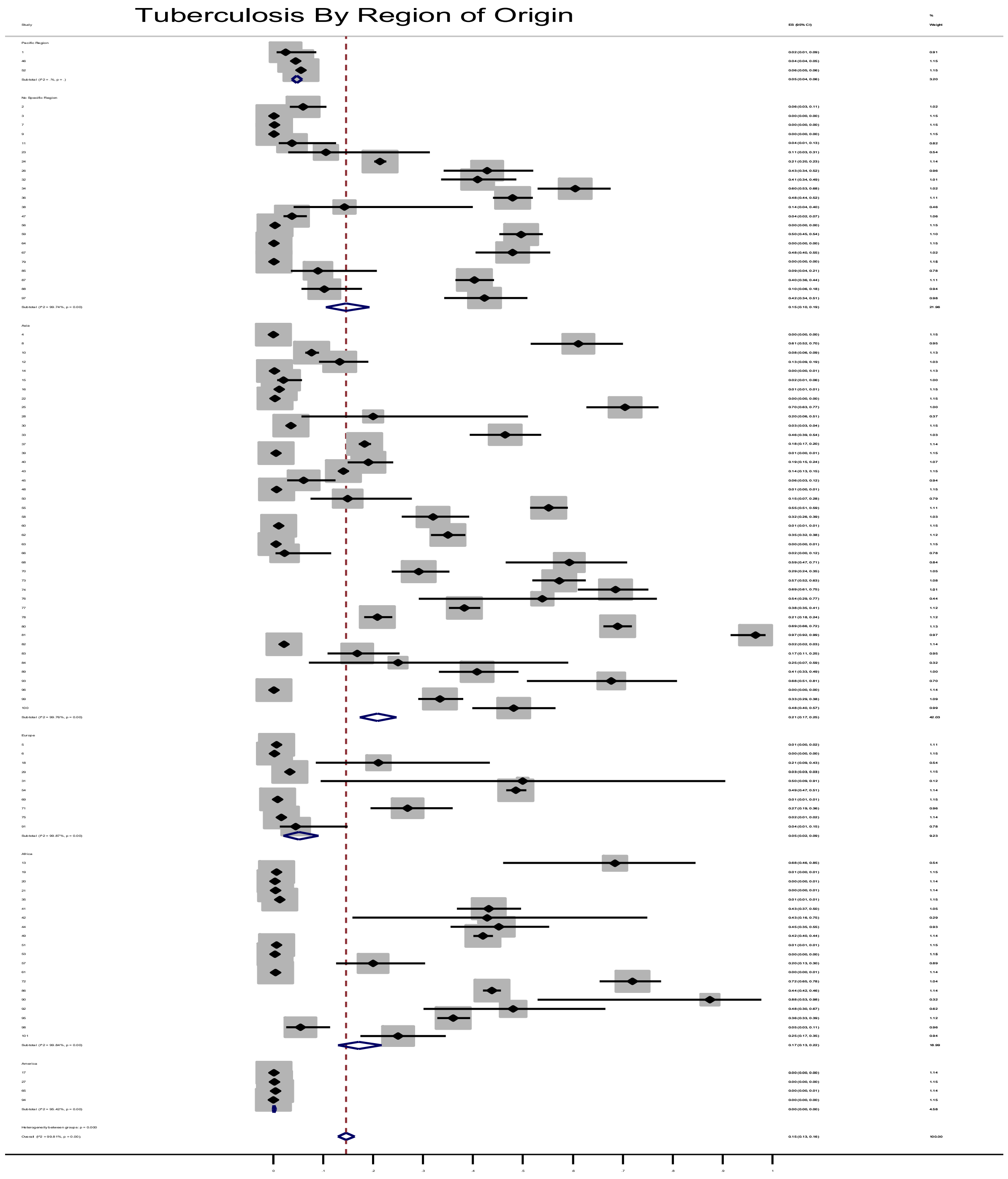

Plot 9.2: Tuberculosis forest plot by host region

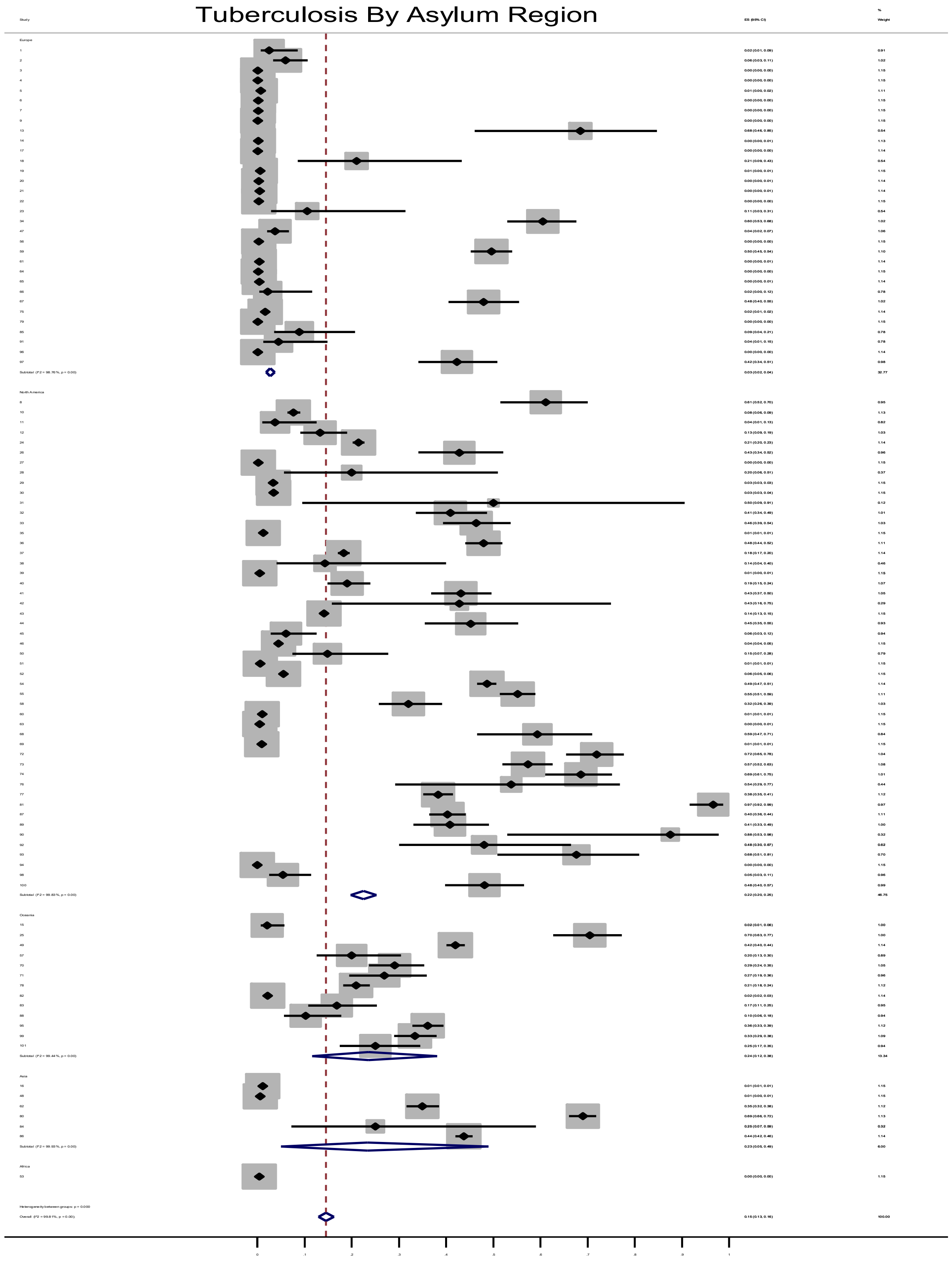

Plot 9.3: Hepatitis B forest plot by Origin region

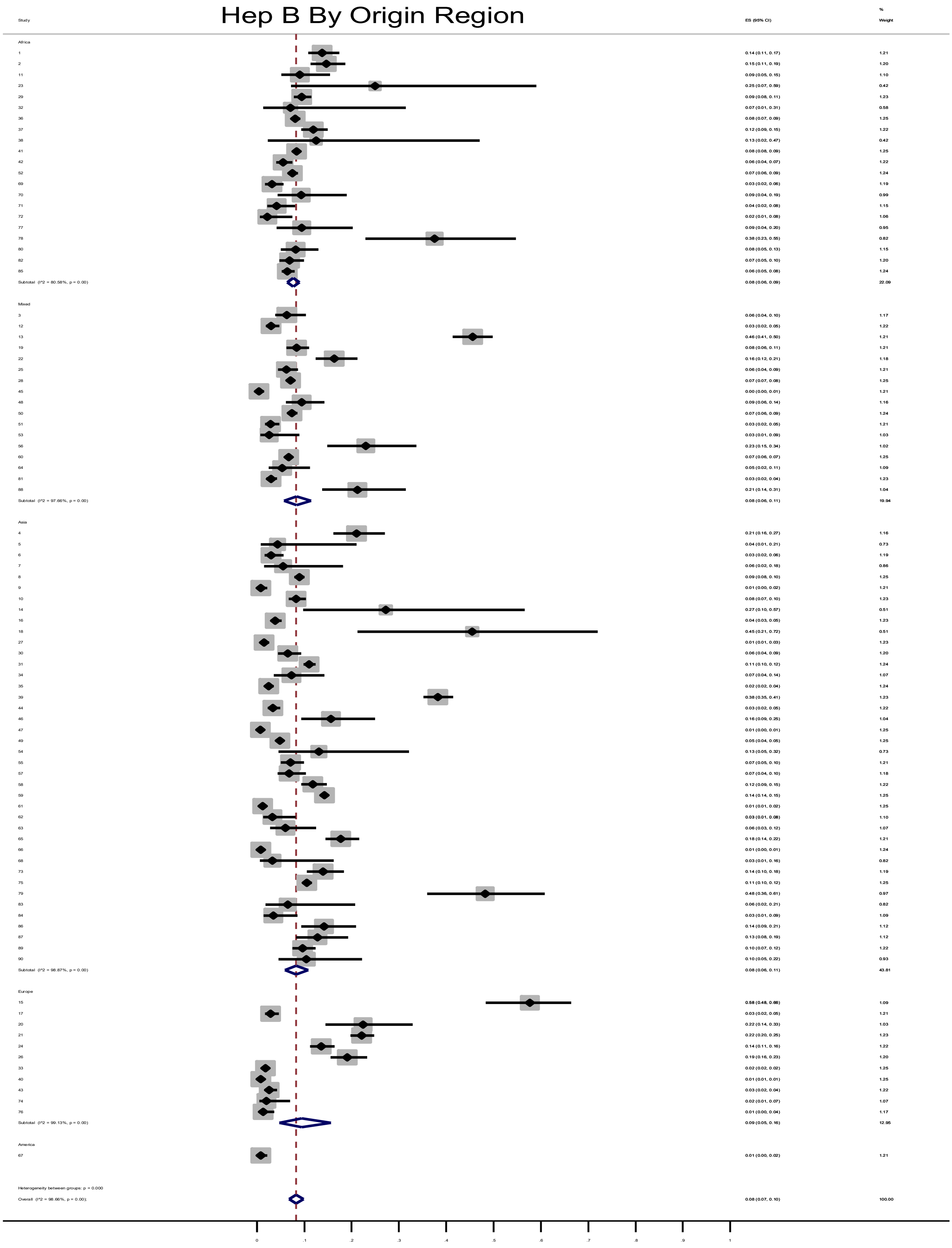

Plot 9.4: Hepatitis B forest plot by Host region

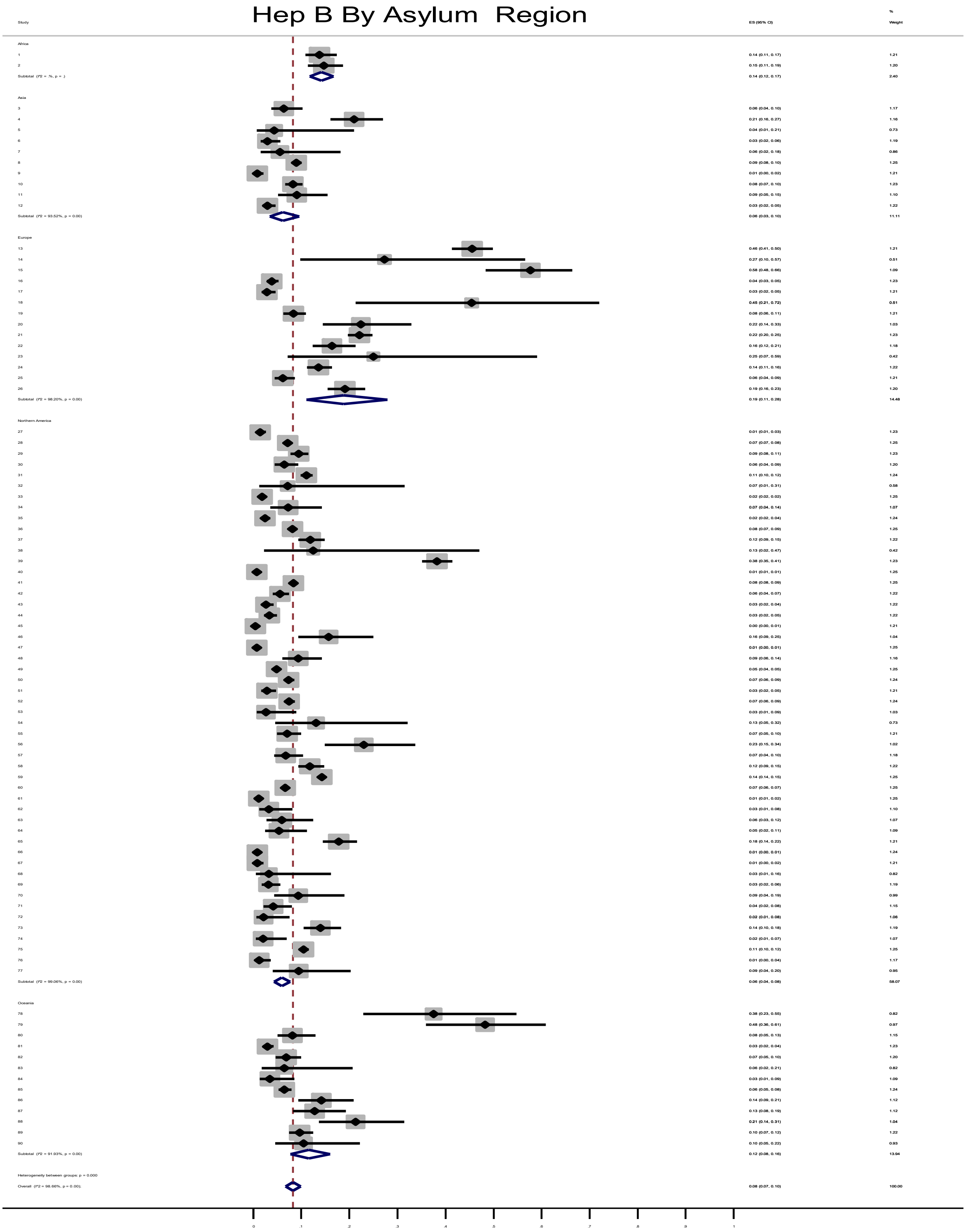

Plot 9.5: Hepatitis C forest plot by Origin region

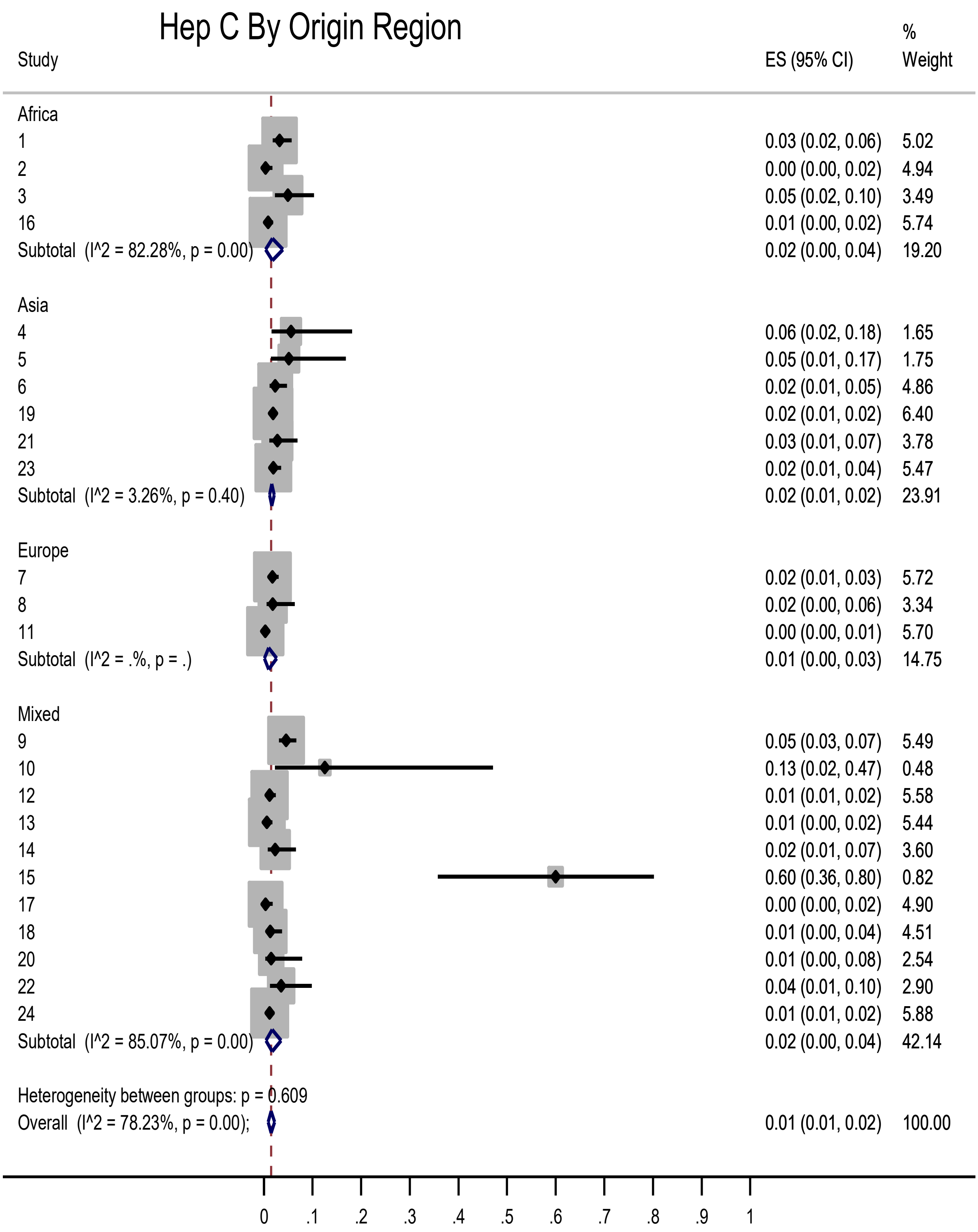

Plot 9.6: Hepatitis C forest plot by Host region

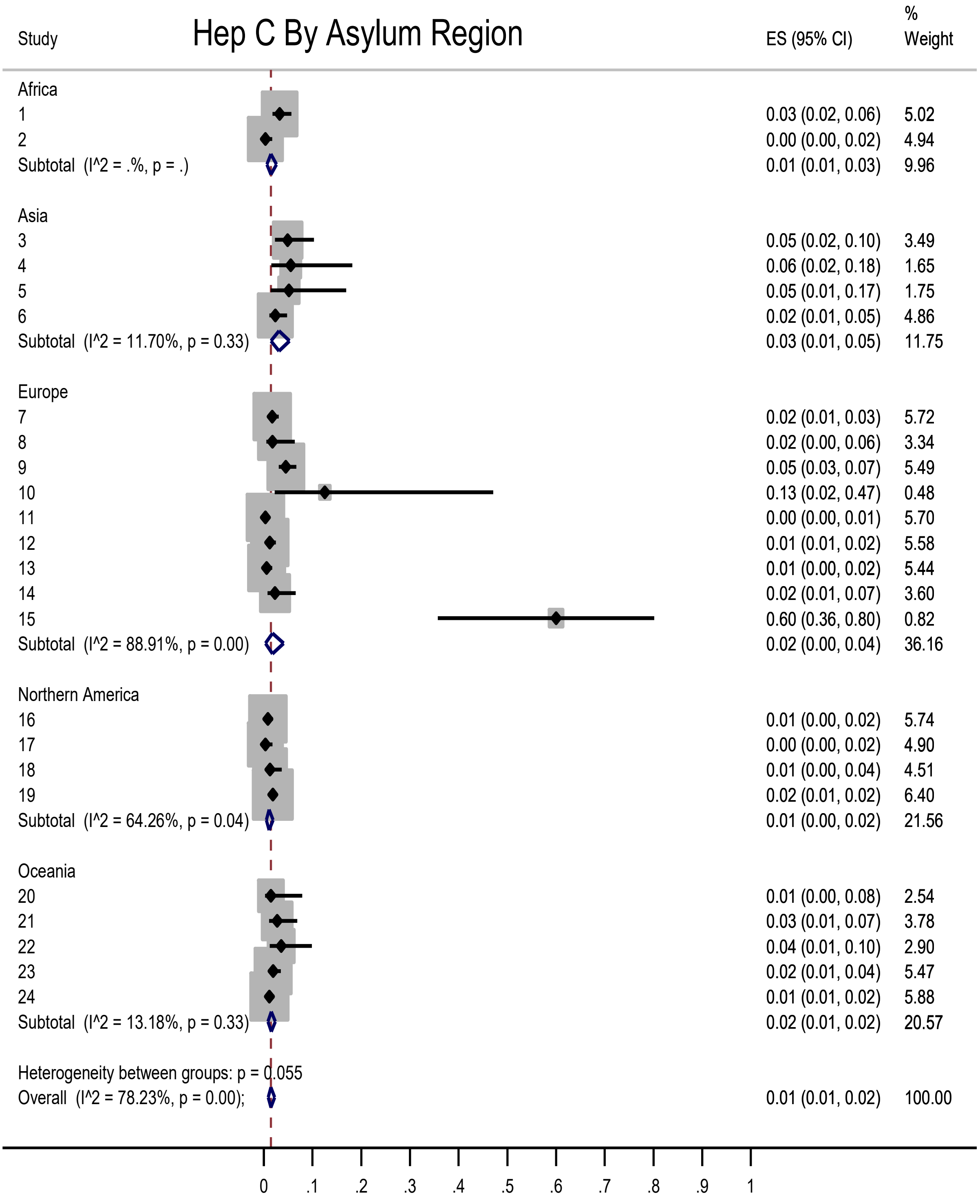
Hep C By Asylum Region
Study
ES (95% CI)
% Weight
Africa
1
0.03 (0.02, 0.06) 5.02
2
0.00 (0.00, 0.02) 4.94
Subtotal (I^2 = .%, p = .)
0.01 (0.01, 0.03) 9.96
Asia
3
0.05 (0.02, 0.10) 3.49
4
0.06 (0.02, 0.18) 1.65
5
0.05 (0.01, 0.17) 1.75
6
0.02 (0.01, 0.05) 4.86
Subtotal (I^2 = 11.70%, p = 0.33)
0.03 (0.01, 0.05) 11.75
Europe
7
0.02 (0.01, 0.03) 5.72
8
0.02 (0.00, 0.06) 3.34
9
0.05 (0.03, 0.07) 5.49
10
0.13 (0.02, 0.47) 0.48
11
0.00 (0.00, 0.01) 5.70
12
0.01 (0.01, 0.02) 5.58
13
0.01 (0.00, 0.02) 5.44
14
0.02 (0.01, 0.07) 3.60
15
0.60 (0.36, 0.80) 0.82
Subtotal (I^2 = 88.91%, p = 0.00)
0.02 (0.00, 0.04) 36.16
Northern America
16
0.01 (0.00, 0.02) 5.74
17
0.00 (0.00, 0.02) 4.90
18
0.01 (0.00, 0.04) 4.51
19
0.02 (0.01, 0.02) 6.40
Subtotal (I^2 = 64.26%, p = 0.04)
0.01 (0.00, 0.02) 21.56
Oceania
20
0.01 (0.00, 0.08) 2.54
21
0.03 (0.01, 0.07) 3.78
22
0.04 (0.01, 0.10) 2.90
23
0.02 (0.01, 0.04) 5.47
24
0.01 (0.01, 0.02) 5.88
Subtotal (I^2 = 13.18%, p = 0.33)
0.02 (0.01, 0.02) 20.57
Heterogeneity between groups: p = 0.055
Overall (I^2 = 78.23%, p = 0.00);
0.01 (0.01, 0.02) 100.00
0
.1
.2
.3
.4
.5
.6
.7
.8
.9
1

Plot 9.7: HIV forest plot by Origin region

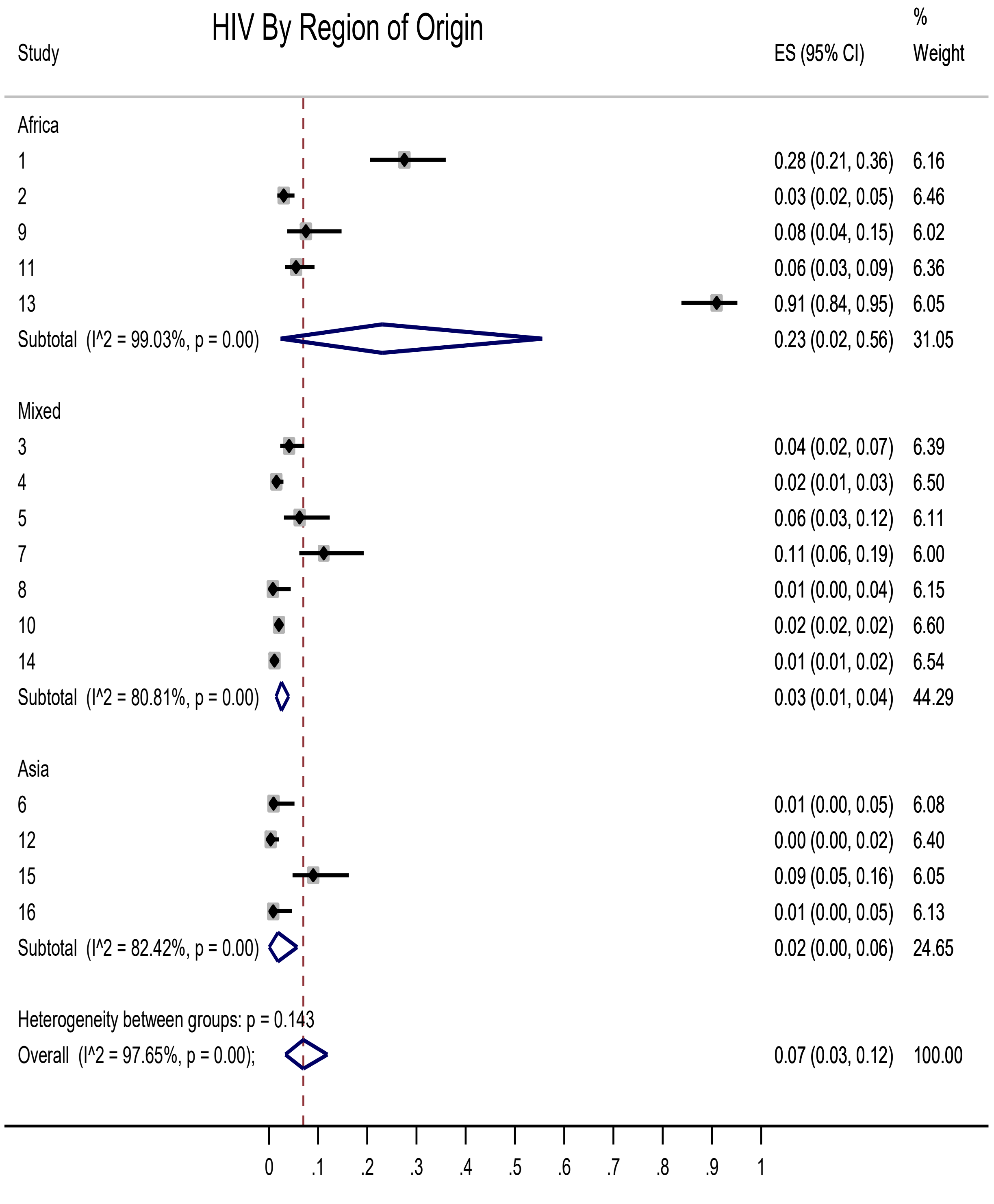

Plot 9.8: HIV forest plot by Host region

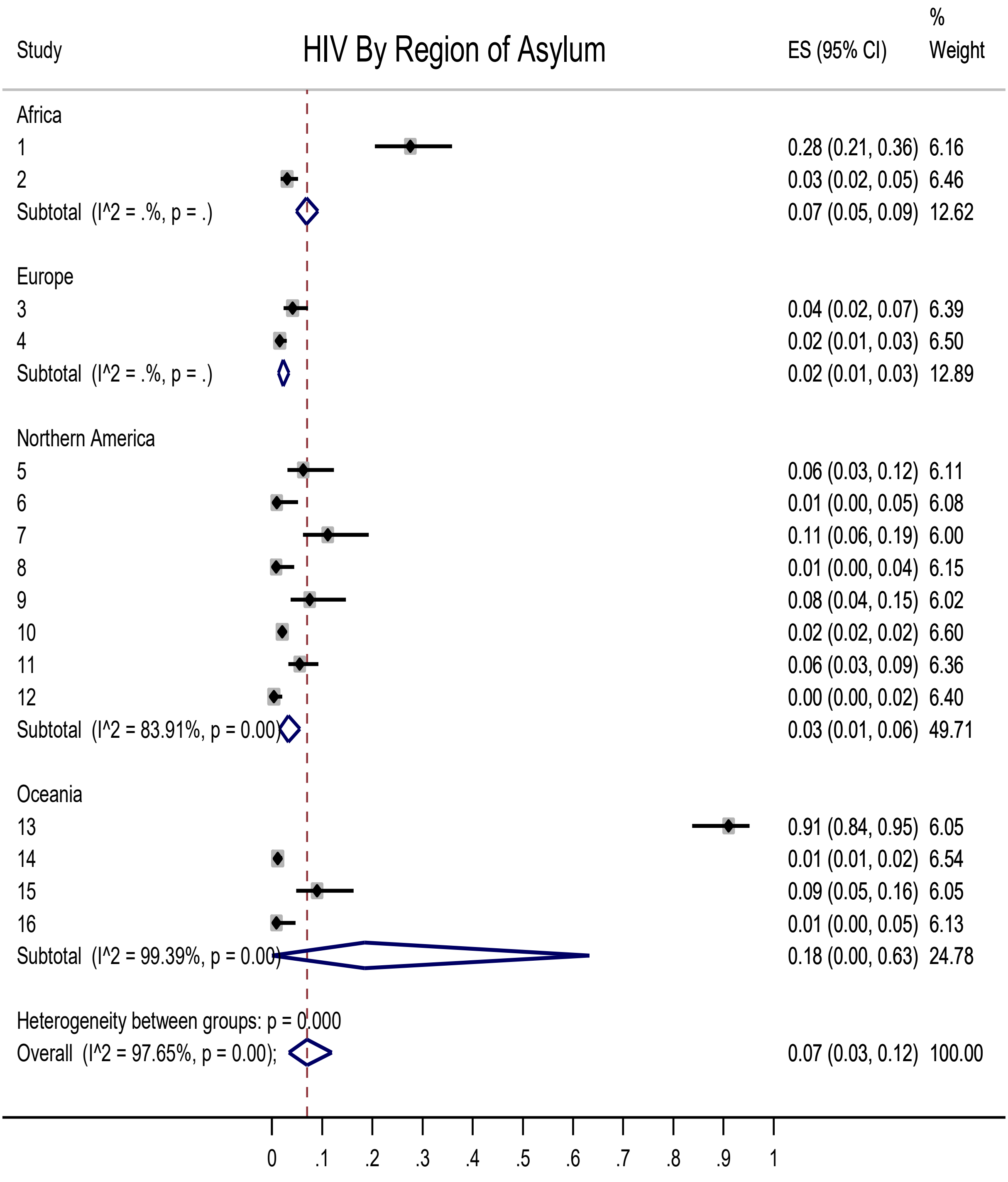
HIV By Region of Asylum
Study
ES (95% CI)
% Weight
Africa
1
0.28 (0.21, 0.36) 6.16
2
0.03 (0.02, 0.05) 6.46
Subtotal (I^2 = .%, p = .)
0.07 (0.05, 0.09) 12.62
Europe
3
0.04 (0.02, 0.07) 6.39
4
0.02 (0.01, 0.03) 6.50
Subtotal (I^2 = .%, p = .)
0.02 (0.01, 0.03) 12.89
Northern America
5
0.06 (0.03, 0.12) 6.11
6
0.01 (0.00, 0.05) 6.08
7
0.11 (0.06, 0.19) 6.00
8
0.01 (0.00, 0.04) 6.15
9
0.08 (0.04, 0.15) 6.02
10
0.02 (0.02, 0.02) 6.60
11
0.06 (0.03, 0.09) 6.36
12
0.00 (0.00, 0.02) 6.40
Subtotal (I^2 = 83.91%, p = 0.00)
0.03 (0.01, 0.06) 49.71
Oceania
13
0.91 (0.84, 0.95) 6.05
14
0.01 (0.01, 0.02) 6.54
15
0.09 (0.05, 0.16) 6.05
16
0.01 (0.00, 0.05) 6.13
Subtotal (I^2 = 99.39%, p = 0.00)
0.18 (0.00, 0.63) 24.78
Heterogeneity between groups: p = 0.000
Overall (I^2 = 97.65%, p = 0.00);
0.07 (0.03, 0.12) 100.00
0
.1
.2
.3
.4
.5
.6
.7
.8
.9
1
